# Adjuvants to improve efficacy of miticides in managed honey bee (*Apis mellifera*) colonies to control *Varroa destructor*

**DOI:** 10.1371/journal.pone.0320037

**Published:** 2025-06-17

**Authors:** Brandon Shannon, Rui Zhang, Lucas Marsh, Reed M. Johnson

**Affiliations:** 1 Department of Entomology, The Ohio State University, Wooster, Ohio, United States of America; 2 Department of Statistics, The Ohio State University, Columbus, Ohio, United States of America; University of Alberta, CANADA

## Abstract

Beekeepers must manage *Varroa destructor* mites to maintain colony health. Large-scale beekeepers often use chemical treatments (miticides) to manage this pest. Miticide resistance drives a need for compounds with alternative modes of toxic action that can be used in rotation as part of a *Varroa* management plan. This research aimed to determine the efficacy of oxalic acid, clove oil, and fenpyroximate when delivered in glycerin soaked in strips and combined with a range of bee-safe adjuvants. Adjuvants are a group of compounds used in plant pesticide applications to increase the spreading and penetration of a pesticide. Laboratory cage trials tested a miticidal active ingredient (oxalic acid, clove oil, or fenpyroximate) and an adjuvant (Ecostep BC-12^®^, Ecostep SE-11^®^, Ecostep AE-13^®^, Ecostep CE-13^®^, or Silwet L-7500^®^) in glycerin-soaked strips. Field trials evaluated the best performing active ingredient-adjuvant combination from cage trials, oxalic acid combined with Ecostep BC-12^®^ adjuvant in glycerin-soaked strips. Neither glycerin control, oxalic acid alone, or oxalic acid with adjuvant caused a significant change in *Varroa* per 100 bees in field trial year 1, when starting *Varroa* levels were high (average 11.8 *Varroa* per 100 bees across all treatment groups). In year 2, when starting *Varroa* levels were low (average 0.58 *Varroa* per 100 bees across all treatment groups), *Varroa* per 100 bees increased 2.6-fold for the glycerin control and 2.8-fold for oxalic acid alone, while a 29% reduction was observed in the oxalic acid with adjuvant treatment. Additionally, mite drop data indicated increased speed for the miticidal effect when an adjuvant is included with oxalic acid. This research informs formulation chemistries for oxalic acid and other miticides to help beekeepers maintain healthy hives.

## Introduction

Honey bees (*Apis mellifera*) are responsible for pollination of at least 100 crops [1–3], with an estimated contribution of 12 billion dollars to the US economy [4,5] and 153 billion USD worldwide [6]. However, high colony losses have been reported by commercial beekeepers, at an annual colony loss rate of 55% and winter loss rate of 37% in the United States [7] and a winter loss rate of 34% in Canada in 2023–2024 [8]. Parasitism by the ectoparasitic mite *Varroa destructor* is a major driver of colony loss. In surveys, 60% of beekeepers self-reported that *Varroa* and its associated diseases were the leading cause of winter colony losses in U.S. commercial beekeeping operations [9,10].

Because many of the non-chemical management strategies for *Varroa* are not sufficiently effective or too labor-intensive [11,12], large-scale beekeepers must use chemical treatments, known as miticides, to manage this pest. However, over-reliance and misuse of synthetic miticides have led *Varroa* to develop worldwide resistance to the formamidine amitraz [13–18], the organophosphate coumaphos [19–22], and the pyrethroids fluvalinate and flumethrin [23–26]. Because of widespread miticide resistance, there is a rising need for products with alternative modes of toxic action that can be used in rotation as part of a *Varroa* management plan. Potential alternatives explored by this study include extended-release formulations of the natural pesticides, oxalic acid and clove oil, and the synthetic pyrazole, fenpyroximate.

Oxalic acid is a miticide that is permitted for use in the United States, Canada, several European countries, and New Zealand [12], and has been used to control *Varroa* for several decades [27]. The mechanism of toxic action of oxalic acid to *Varroa* is not well understood [28], though effects of physical damage to the chitin plate of *Varroa* have been observed [29]. However, oxalic acid is not effective against *Varroa* protected by brood cell cappings, so it has limited efficacy when applied as a vaporization or dribble flash treatment when brood is present [30,31]. At the time of this study, no oxalic acid extended-release treatments were available for use in the United States, but many beekeepers treated with off-label oxalic acid extended release formulations using sponges or shop towels soaked in a 1:1 w/w mixture of glycerin and oxalic acid [32]. While clove oil (principal constituent eugenol) does not have a defined mode of action, it has demonstrated contact toxicity to *Varroa* [33–36]. Clove oil can act as an insect repellent and prohibits the growth of bacteria and fungi [37], but likely affects *Varroa* through effects on the enzymes glutathione-S-transferase, superoxide dismutase, and Ca^2+^-Mg^2+^-ATPase [38]. Fenpyroximate is a synthetic pyrazole acaricide in IRAC class 21A Mitochondrial Complex I Electron Transport Inhibitor [39] that was previously registered for in-hive use as Hivastan^®^ and demonstrates promising mite control in semi-field evaluations [40]. Oxalic acid is registered for use as a dribble or vaporization treatment as Api-Bioxal^®^, as a vaporization treatment as EZ Ox^®^, or as an extended-release strip formulation as Varroxsan^®^ [41], though only Api-Bioxal^®^ was registered for use at the start of this experiment. Neither clove oil nor fenpyroximate are currently formulated in registered *Varroa* control products.

Adjuvants are added to pesticides, either as formulation components or tank-mix components, to improve the handling or application characteristics and enhance pesticide activity [42]. While adjuvants are typically used with spray applications in agriculture, they may also be included in some pesticides used to control common bee pests in managed honey bee colonies [43], though the identity of adjuvant ingredients in these products are proprietary. The “principal functioning agents” that provide the desired function of an adjuvant are drawn from the list of inert ingredients maintained by US EPA and consist of the same or similar compounds used as formulation components in traditional pesticide products [44]. While some adjuvants have shown high toxicity to honey bees, others are relatively non-toxic [43,45]. Adjuvants are widely used and were valued globally at 3.8 billion USD in 2023 [46], but we could find no published studies that sought to make use of bee-safe adjuvants to improve pesticide efficacy within honey bee colonies. Using adjuvants that demonstrate low toxicity to bees as formulation components in *Varroa* control applications can potentially improve miticide activity and increase the number of active ingredients available for control of *Varroa* mites [47].

Oxalic acid dihydrate, clove oil, and fenpyroximate have shown promise as miticides for *Varroa* control, but demonstrate limited efficacy at concentrations that are safe for honey bees. This research aimed to determine the efficacy of these active ingredients when delivered in glycerin soaked strips and combined with a range of bee-safe adjuvants. Combinations of active ingredients and adjuvant were first tested in laboratory cage trials to identify promising miticide-adjuvant combinations. The most promising combination, oxalic acid active ingredient with Ecostep BC-12 adjuvant, was then assessed in field trials to determine if the addition of an adjuvant could improve *Varroa* control in whole honey bee colonies.

## Materials and methods

### Miticides and adjuvants

The miticides used in the laboratory cage trial included clove oil (100%; Sigma-Aldrich; MO, USA); fenpyroximate (>98.0%; Alfa Chemistry, Inc.; NY, USA); and oxalic acid dihydrate (99.5–102.5%; Thermo Fisher Sceintific; MA, USA). The miticide used in the field trial was oxalic acid dihydrate.

The adjuvants used in the laboratory cage trials are listed in Table 1 and include Ecostep AE-13^®^ (Stepan, Northfield, IL, USA), Ecostep BC-12^®^ (Stepan), Ecostep CE-13^®^ (Stepan), Ecostep SE-11^®^ (Stepan), and Silwet L-7500 Copolymer^®^ (Momentive Performance Materials, Niskayuna, NY, USA). All four Ecostep products are listed for organic use through the Organic Materials Review Instritute (OMRI). The principal functioning agents for all adjuvants are included in the list of inert ingredients safe for food and nonfood use [44]. The adjuvant used in the field trial was Ecostep BC-12^®^.

**Table 1:** Adjuvant treatments used in this experiment. An asterisk (*) indicates that the concentration of principal functioning agents is not given and the SDS states that the components are not hazardous or are below required disclosure limits.

Treatment	Manufacturer	Principal Functioning Agents (% Composition)
Ecostep AE-13^®^	Stepan	Polyethylene Glycol/ Polypropylene Glycol – (n/n) Copolymer (> 99%)
Ecostep BC-12^®^	Stepan	Polyethylene/ Polypropylene Glycol Monobutyl Ether (90–100%)
Ecostep CE-13^®^	Stepan	Castor Oil, Ethoxylated (90–100%)
Ecostep SE-11^®^	Stepan	Sorbitan, Mono-(9z)-octadecenoate, Poly(oxy-1,2-ethanediyl) Derivs (90–100%)
Silwet L-7500 Copolymer^®^	Momentive Performance Materials	Polyalkyleneoxide modified polydimethylsiloxane*

### Adjuvant acute toxicity tests

All adjuvants were tested for contact bee toxicity using a Potter Spray Tower [48]. The insecticide Mustang Maxx^®^ (9.15% zeta-cypermethrin active ingredient), a pyrethroid insecticide with high acute toxicity to honey bees, was used as a positive control [45,49–51]. The 48-hour acute contact LC_50_ of all adjuvants were determined using 3-day-old adult worker bees following methods described in [45]. Data analysis was performed with the *drc* package in R [52] using 2-parameter log-logistic dose-response models, as performed by Shannon et al. [45]. The concentrations tested were as follows: for the four Ecostep^®^ products, 0, 1, 3, 5, 10, and 20% by volume; for Silwet L-7500 Copolymer^®^, 0, 0.3, 1, 3, 5, 10, and 20% by volume; for Mustang Maxx^®^ Positive Control, 0.00186, 0.00558, 0.0186, 0.0558, and 0.186% by volume of formulation, which is 1.70e-4, 5.10e-4, 1.70e-3, 5.10e-3, 1.70e-2% zeta-cypermethrin active ingredient by volume [45,51].

### Cage trials

#### Treatment preparation.

Treatment solutions were prepared by adding glycerin (Fisher Scientific; MA, USA) to a 15 mL conical tube (Thermo Fisher Scientific; MA, USA) and sonicating with heating (Kendal Digital Ultrasonic Heated Cleaner model HB-S-49DHT) for 30 min to make 3 g of final treatment mixture. Negative control treatment solutions containing only glycerin were also heated. Clove oil treatments were prepared by adding clove oil to form a 3% v/w mixture with heating at 34º C. Fenpyroximate treatments were prepared by first dissolving solid fenpyroximate in acetone to a 10% w/v solution, then adding the fenpyroximate-acetone solution to glycerin to produce a 0.2% w/w solution that was heated to 34º C. Oxalic acid treatments were prepared by adding oxalic acid dihydrate to glycerin, up to, but not above, 67º C [53–56] and sonication for a minimum of 30 minutes, until crystals were no longer visible, to produce a 20% w/w solution. The concentrations of the active ingredients in cage trials were chosen based on preliminary studies that determined the maximum concentrations that could be applied with minimal mortality observed in caged bees.

Active ingredient with adjuvant combination treatments were prepared by adding a single adjuvant product (Ecostep AE-13^®^, Ecostep BC-12^®^, Ecostep CE-13^®^, Ecostep SE-11^®^, or Silwet L-7500 Copolymer^®^) to the glycerin-active ingredient mixture and mixed fully to produce a 0.5% w/w solution for the Ecostep products or a 0.2% w/w solution for Silwet L-7500 Copolymer. The final active ingredient concentration was the same as in the active ingredient controls. The concentration of adjuvant was chosen based on the labeled concentrations for similar adjuvants in agricultural tank mix applications, which typically range from 0.0625 to 0.625 percent by volume [45].

Cage treatment strips were made from a Swedish sponge (Superscandi Swedish Dishcloths; London, England) cut into 32 sections (1.25 x 8.5 cm). A single treatment strip was placed in each 3 g solution and heated with sonication at the respective temperature for each treatment for 30 minutes. The concentration of 1/32 saturated treatment strip was used in cages to scale down a full-size strip that would be used with 10,000 bees in a deep Langstroth box [57,58] to approximately 300 bees in each cage.

#### Cage design.

The design of cages above petroleum jelly-coated weigh boats was modified from Rinkevich, 2020 [17] (S1 Text, Fig A). Modifications include the following: a 15 mm x 3 mm slit cut in the top of the cage to insert the 1.25 x 8.5 cm treatment strip; four equally spaced 3-mm holes were added for airflow on the sides of the cage, 1 cm from the top; two 3–5 g sucrose cubes were fixed to the top of the cage using hot melt adhesive to allow bees to feed.

#### Honey bees.

To populate the cages, honey bees were shaken from brood frames from six colonies, in July through October in 2022 and 2023. Colonies were managed at The Ohio State University – Wooster campus apiaries and had not been treated for mites for at least 6 months prior to collection. All colonies had been requeened with New World Carniolan queens (Olivarez Honey Bees, Orland, CA) in the Spring, but queens were caged for at least 21 days prior to worker bee collection to maximize the number of mites in the dispersal phase. Bees were shaken into an empty 4-frame nucleus box and stored in darkness for no longer than one hour before transferring to cages. Bees were misted with DI water during transfer to discourage flight. Approximately 300 bees were scooped from the nucleus box and placed into each cage containing a treatment.

#### Experimental design.

Each cage treatment series consisted of 6 treatments: a negative control (glycerin solvent only), active ingredient control (active ingredient in glycerin), and four combinations of that same active ingredient in glycerin with four different adjuvants. Using a cage assay rather than a traditional topical application or glass vial application better simulates the exposure that mites would face in a colony environment, but may introduce additional sources of variability compared to other topical application methods [59], from variable age [60–62], *Varroa* loads [59], bee health [12,63,64], and colony strength [65]. This variability was managed by performing each set of treatments, assigned in random order, with the same cohort of bees. A minimum of 3 replicates were performed for each treatment. Cages were placed inside an incubator (Humidaire Model No. 2048; The Humidaire Incubator Company, New Madison, OH, USA) and stored at hive conditions (34º C, 60% humidity, darkness). Dead bees at the bottom of the cage and dead *Varroa* on the collection tray were counted after 24 hours. After bees and *Varroa* were counted, the plastic tray was discarded and cages were inverted and frozen at −20º C.

Bees were weighed (Ohaus, Parsippany, NJ; Model CL5000) and the number of grams was multiplied by 11.34 to estimate the number of bees, as determined in preliminary trials. Bees were agitated for 30 minutes in 70% ethanol to dislodge *Varroa* remaining on the bees for counting [66,67]. Recovery efficacy of alcohol washes was not evaluated, but the same methods were used for all samples. Treatment efficacy was determined by dividing the number of *Varroa* that had fallen during treatment by the total number of *Varroa* (mites fallen plus mites in alcohol wash)*.* A logistic regression was used to determine significant differences in bee mortality and *Varroa* control efficacy [68], followed by a Tukey HSD post-hoc test [69].

### Field trials

#### Honey bee colonies.

Three apiaries, separated by a minimum of 5 km, located at The Ohio State University – Wooster campus, were used to conduct the field trial. Each colony consisted of a minimum of two deep 8-frame Langstroth boxes at the start of the experiment. In year 1, each colony was fitted with a screened bottom board for collecting mite drop data. In year 1, 9 hives from apiary 1, and 6 hives each from apiaries 2 and 3 (21 total) were randomly assigned treatments. In year 2, 9 hives from each of the 3 apiaries (27 total) were randomly assigned treatments. In both years, treatments were stratified based on pre-treatment alcohol wash *Varroa* levels and were assigned so that each apiary had an equal number of replicates of each treatment.

#### Experimental design.

Before and after treatment, colonies were assessed by performing *Varroa* alcohol washes and seam counts [70–72]. Seam counts are used to estimate the number of adult bees in a colony and involve two observers visually estimating the clusters or “seams” of bees found between the frames in each box of the hive. Seams in medium boxes were multiplied by the height of a medium Langstroth box divided by the height of a deep Langstroth box (6.625/9.625). Any colonies with less than 9 seams prior to treatment or less than 2 seams after the treatment were excluded from analysis. Alcohol washes were performed by collecting approximately 300 bees from 3 worker brood frames into 70% ethanol, followed by shaking 30 minutes, and counting both bees and *Varroa* that were strained from the wash.

For year 1, *Varroa* that fell below the colony during the treatment, or mite drop, was monitored at 48-hour intervals starting 2 days prior to treatment, at the time of treatment application (day 0), and then 2, 4, 7, 14, and 21 days following treatment. Mite drop was monitored by placing open letter-sized (43.2 cm x 27.9 cm) manila file folders (Staples, Framingham, MA, USA) coated with Vaseline^®^ petroleum jelly (Unilever, Egewood Cliffs, NJ, USA) under screened bottom boards. After folders were removed, they were folded closed and frozen at −20º C for a minimum of 24 hours and stored under these conditions until counted.

#### Treatments.

The combination of oxalic acid active ingredient with Ecostep BC-12^®^ adjuvant was chosen for field trials as it had the highest efficacy in cage trials. In year 1, the adjuvant combination treatment consisted of 1% w/w Ecostep BC-12 adjuvant and 40% w/w oxalic acid dihydrate dissolved in glycerin, the oxalic acid control consisted of 40% w/w oxalic acid dihydrate dissolved in glycerin, and the glycerin control consisted of glycerin only. In year 2, a 0.5% w/w adjuvant concentration was used instead of the 1% concentration in the oxalic acid plus adjuvant treatment. For each uncut Swedish sponge, 70 g of solution, the amount needed for complete saturation of one sponge, was prepared and heated at less than 67º C for at least 1 hour with sonication (Kendal Digital Ultrasonic Heated Cleaner model HB-S-49DHT) and stirring, if necessary.

In year 1, one treatment strip, consisting of a full Swedish sponge (20.3 x 17.8 cm) saturated in treatment solution, was applied for every 9 seams of bees, rounded up, so that each colony had either 2 or 3 treatment strips. In year 2, Swedish sponges were cut in half to increase contact area with bees, and strips were applied for every 5 seams of bees, rounded up, so that each colony had at least 3 treatment strips. Treatment strips were placed between boxes containing brood. Colonies were left undisturbed during the treatment period. In year 1, treatments were in place for 23 days starting on Sept 21, 2023 for apiary 1, Oct 9 for apiary 2, and Oct 14 for apiary 3. In year 2, treatments were applied for 22 days starting on July 30, 2024 for all apiaries.

#### Data analysis.

Field trial results were analyzed independently for each year using the same statistical analysis methods. Efficacy within treatments was compared by modelling individual wash counts using a generalized linear mixed model (GLMM) with a negative binomial distribution and a random intercept for colony [73]. To analyze cumulative mite drop over the full 23-day treatment period in year 1, we modeled the data using a negative binomial generalized linear model [74] to account for overdispersion in count data (S2 Text). The model included treatment and used forward selection to choose variables for inclusion in the model, which included the following predictors: apiary, initial seam count, initial mite wash, and baseline mite drop. A second negative binomial generalized linear model with the same predictors was used for analyzing cumulative mite drop counts over the first 2 and 4 days of treatment.

## Results

### Screening adjuvant toxicity to bees through a spray application

The 48-hour LC_50_ of all adjuvants tested was predicted to be greater than the maximum concentration sprayed with the Potter Tower (Table 2; S3 Dataset), and the mean mortality at the highest tested concentration for each adjuvant was below 50%. The LC_50_ for Mustang Maxx^®^, the positive control, was estimated to be 0.043%, (95% CI = 0.038, 0.047) when expressed as a formulation concentration, or 0.0039% (95% CI = 0.0035, 0.0044) when expressed as a concentration of zeta-cypermethrin active ingredient.

**Table 2:** 48-hour LC_50_ estimates of each adjuvant applied to adult bees using a Potter Spray Tower. All adjuvant LC_50_ estimates were above the maximum rate tested. Values in parenthesis indicate 95% confidence intervals.

Adjuvant	Concentrations tested (% v/v)	48-hour LC50 (% v/v)	Slope (–b)
Ecostep AE-13^®^	1, 3, 5, 10, 20	> 20	0.46 (0.16, 0.76)
Ecostep BC-12^®^	1, 3, 5, 10, 20	> 20	1.07 (0.71, 1.42)
Ecostep CE-13^®^	1, 3, 5, 10, 20	> 20	0.40 (0.15, 0.66)
Ecostep SE-11^®^	1, 3, 5, 10, 20	> 20	0.93 (0.60, 1.25)
Silwet L-7500 Copolymer^®^	0.3, 1, 3, 5, 10, 20	> 20	0.65 (0.25, 1.04)
Zeta-cypermethrin Positive Control	1.70e-4, 5.10e-4, 1.70e-3, 5.10e-3, 1.70e-2	0.0039 (0.0035–0.0044)	2.0 (1.7, 2.4)

### Cage trials

#### Clove oil.

**Fig 1:**
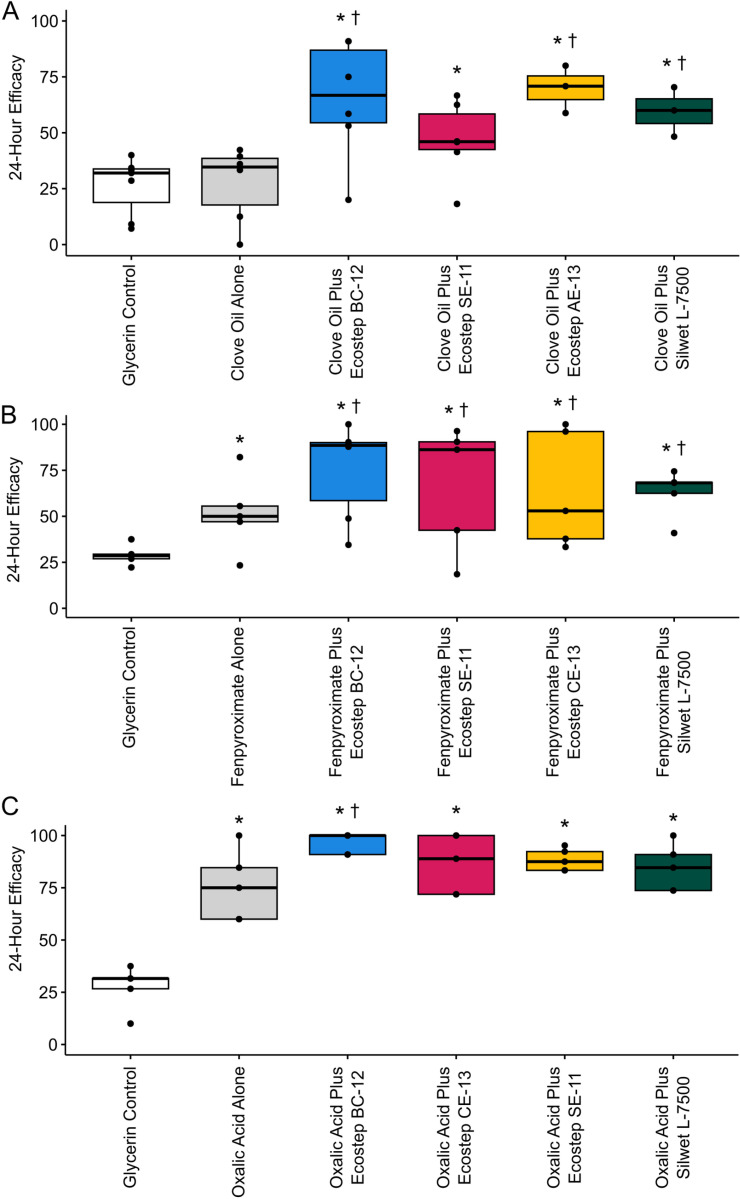
Efficacy of 3% Clove Oil **(A), 0.2% Fenpyroximate (B), and 20% Oxalic Acid (C) treatments in cage trials,** where efficacy is determined to be the number of Varroa mites that fell in 24 hours divided by the total number of Varroa mites in each cage. Adjuvant concentrations were 0.5% for all Ecostep adjuvants and 0.1% for Silwet L-7500 copolymer. Significant difference (P < 0.05) from the glycerin control is indicated by an asterisk (*) and significant difference (P < 0.05) from the active ingredient alone is indicated by a dagger (†).

There was a significant difference in 24-hour bee mortality among treatments (logistic regression, P = 0.028; S1 Text, Table A; S4 Dataset). Tukey’s post-hoc test determined that Ecostep BC-12 with clove oil caused significantly more bee mortality than the glycerin control (P = 0.050). A significant difference was observed in 24-hour *Varroa* efficacy (logistic regression, P < 0.001; Fig 1A; S1 Text, Table A; S4 Dataset). Tukey’s post-hoc test determined that Ecostep AE-13, Ecostep BC-12, Ecostep SE-11, and Silwet L-7500 Copolymer with clove oil caused significantly increased *Varroa* efficacy (P < 0.05) compared to the glycerin control, and that Ecostep AE-13, Ecostep BC-12, and Silwet L-7500 Copolymer with clove oil caused significantly increased *Varroa* efficacy (P < 0.05) compared to the clove oil alone.

#### Fenpyroximate.

There was a significant difference in 24-hour bee mortality among treatments (logistic regression, P < 0.001; S1 Text, Table A; S4 Dataset). Tukey’s post-hoc test determined significantly increased bee mortality (P < 0.05) for Ecostep AE-13, Ecostep BC-12, and Silwet L-7500 Copolymer with fenpyroximate compared to the glycerin control. A significant difference was observed in 24-hour *Varroa* efficacy (logistic regression, P < 0.001; Fig 1B; S1 Text, Table A; S4 Dataset). Tukey’s post-hoc test determined that fenpyroximate alone, as well as Ecostep BC-12, Ecostep CE-13, Ecostep SE-11, and Silwet L-7500 Copolymer with fenpyroximate caused significantly increased *Varroa* efficacy (P < 0.05) compared to the glycerin control, and that Ecostep BC-12, Ecostep CE-13, Ecostep SE-11, and Silwet L-7500 Copolymer with fenpyroximate caused significantly increased *Varroa* efficacy (P < 0.05) compared to the fenpyroximate alone.

#### Oxalic acid.

There was a significant difference in 24-hour bee mortality among treatments (logistic regression, P = 0.018; S1 Text, Table A; S4 Dataset). However, Tukey’s post-hoc test determined no significant differences in bee mortality between any of the treatments. A significant difference was observed in 24-hour *Varroa* efficacy (logistic regression, P < 0.001; Fig 1C; S1 Text, Table A; S4 Dataset). Tukey’s post-hoc test determined that oxalic acid alone, as well as Ecostep BC-12, Ecostep CE-13, Ecostep SE-11, and Silwet L-7500 Copolymer with oxalic acid caused significantly increased *Varroa* efficacy (P < 0.05) compared to the glycerin control, and that only Ecostep BC-12 caused significantly increased *Varroa* efficacy (P < 0.05) compared to the oxalic acid alone. The combination of oxalic acid with Ecostep BC-12 was the most efficacious treatment of all active ingredient-adjuvant combinations, with an efficacy of 96.4% (standard deviation = 5.0).

### Field trials

Colonies that had 2 or fewer seams of bees by the end of the experiment were excluded from analysis. In year 1, the analysis included 5 colonies from the glycerin control group, 7 colonies from the oxalic acid control group, and 6 colonies from the oxalic acid plus adjuvant treatment group. In year 2, no colonies were excluded and all treatment groups included 9 colonies.

#### Pre- and post-treatment ethanol washes.

**Fig 2:**
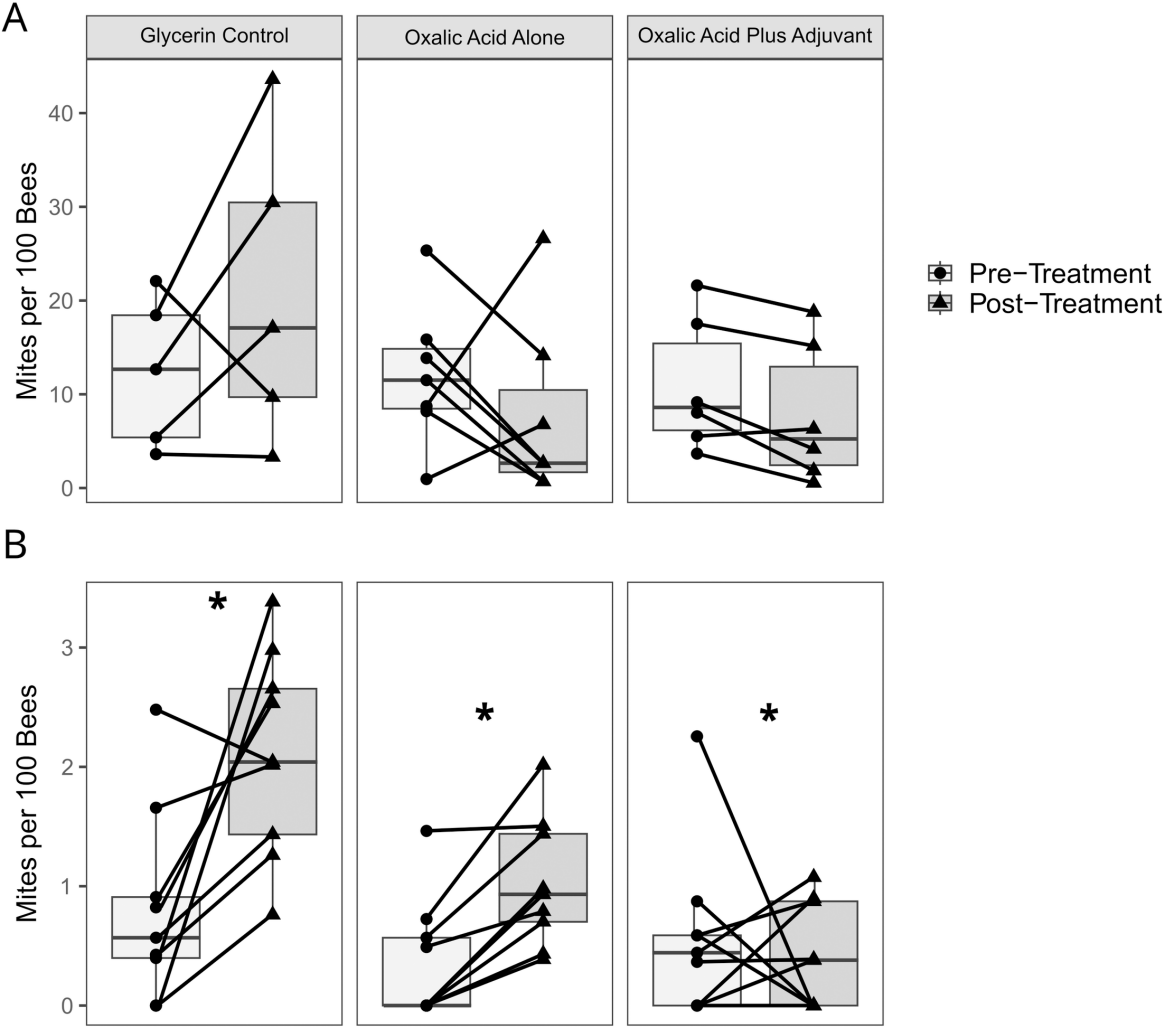
*Varroa* levels from pre- and post-treatment alcohol washes in year 1 (A) and year 2 (B) field trials. Points indicate mites per 100 bees for each colony, and lines connect pre- and post-treatment data for each individual colony. A statistically significant difference (P < 0.05) between post- and pre-treatment *Varroa* levels within treatments, determined using a GLMM, is indicated with an asterisk (*).

In year 1, the glycerin control colonies had a 1.6-fold increase in *Varroa* per 100 bees (Fig 2A; S1 Text, Table B, Fig B; S5 Dataset), an average increase of 8.4 *Varroa* per 100 bees (95% CI = 1.7, 15.1), from 12.4 (SD = 7.1) to 20.8 (SD = 16.3). However, this increase was not statistically significant (z = 1.203, P > 0.1). The oxalic acid alone had a 43.4% reduction in *Varroa* per 100 bees, which was an average decrease of 4.3 *Varroa* per 100 bees (95% CI = −8.7, 0.1), from 12.1 (SD = 7.6) to 7.6 (SD = 9.6). This decrease was not statistically significant (z = −1.576, P > 0.1). A 35.2% reduction in *Varroa* per 100 bees was observed between the pre- and post-treatment ethanol washes for the oxalic acid plus adjuvant treatment, which was an average decrease of 3.1 *Varroa* per 100 bees (95% CI = −4.1, −2.1), from 10.9 (SD = 7.1) to 7.8 (SD = 7.5). This decrease was also not statistically significant (z = −1.155, P > 0.1).

In year 2, a 2.6-fold increase in *Varroa* per 100 bees was observed for colonies in the glycerin control (Fig 2B; S1 Text, Table B, Fig B; S5 Dataset), an average increase of 1.3 *Varroa* per 100 bees (95% CI = 0.9, 1.7), from 0.8 (SD = 0.8) to 2.1 (SD = 0.9). This increase was statistically significant (z = 22.020, p < 0.0001). In the oxalic acid alone treatment, *Varroa* per 100 bees increased 2.8-fold after treatment, an average increase of 0.7 *Varroa* per 100 bees (95% CI = 0.5, 0.8), from 0.4 (SD = 0.5) to 1.0 (SD = 0.5). This increase was statistically significant (z = 16.102, p < 0.0001). In the oxalic acid plus adjuvant treatment, a 29% reduction in *Varroa* per 100 bees was observed between the pre- and post-treatment ethanol washes, which was an average decrease of 0.2 *Varroa* per 100 bees (95% CI = −0.5, 0.2), from 0.6 (SD = 0.7) to 0.4 (SD = 0.4). This decrease was statistically significant (z = −5.016, P < 0.0001).

#### Varroa mite drop.

**Fig 3:**
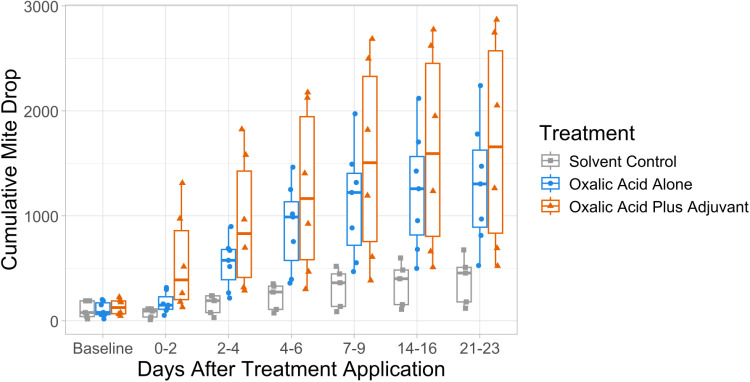
The cumulative mite drop for colonies in Year 1. Each point indicates the average daily mite drop for each measurement period plus the sum of all previous measurement periods. The baseline measurement is not included in the cumulative measurement. Treatments were applied at day 0, immediately prior to the start of mite drop sample collection.

Over the 23-day treatment period in year 1, colonies treated with oxalic acid with adjuvant had a 4.7-fold increase in cumulative mite drop compared to the glycerin control (p < 0.001; Table 3; Fig 3; S5 Dataset). In the first 4 days, colonies treated with oxalic acid with adjuvant had a 6.4-fold increase in total mite drop compared to the glycerin control (p < 0.001), and a 1.46-fold increased total mite drop compared to oxalic acid alone, but this difference was not statistically significant (p = 0.117). In the first 2 days, colonies treated with adjuvant had an 8.5-fold increase in mite drop compared to the glycerin control (P < 0.001), and a 2.8-fold increase in mite drop compared to oxalic acid alone, which was statistically significant (P < 0.001).

**Table 3:** Average daily *Varroa* mite drop over the treatment period. Day 0 is the initial time of treatment. Baseline indicates daily mite drop for the 48-hour period prior to treatment application. Values indicate average daily mite drop during the measurement period for each treatment, with standard deviation listed in parenthesis, where baseline indicates the 48-hour period before treatment.

Days after treatment	Baseline	0-2	2-4	4-6	7-9	14-16	21-23
Glycerin control	103 (82.0)	73.5 (47.1)	81.9 (48.2)	72.5 (34.8)	82.4 (63.5)	38.9 (24.5)	38.4 (26.8)
Oxalic Acid Control	109 (72.1)	171 (99.8)	377 (160)	342 (189)	240 (140)	104 (65.1)	66.3 (45.4)
Oxalic Acid Plus Adjuvant	130 (76.8)	563 (481)	383 (194)	288 (195)	298 (176)	94.0 (38.7)	65.1 (47.4)

## Discussion

Ecostep BC-12 adjuvant increased the efficacy of oxalic acid in cage trials without any increase in honey bee mortality, with an average efficacy exceeding 96%. In the field trial in year 1, neither oxalic acid alone nor oxalic acid combined with Ecostep BC-12 resulted in a statistically significant reduction in *Varroa* measured with an alcohol wash. The control group also showed no increase in *Varroa* per 100 bees over the course of the experiment. In the field trial in year 2, the results were more definitive: colonies treated with oxalic acid alone experienced a 184% increase in mite counts following treatment, indicating that oxalic acid without an adjuvant was ineffective under these field conditions. In contrast, colonies treated with oxalic acid combined with Ecostep BC-12 experienced a 29% reduction in mite loads, demonstrating clear efficacy when the adjuvant was included. While oxalic acid extended-release formulations in glycerin have been found to be effective in some studies [75–78], other studies found them to be ineffective [79]. No significant changes in mite levels were observed for any of the three treatments in year 1, when pre-treatment *Varroa* levels were high (average 11.8 mites per 100 bees across all treatment groups). In year 2, when pre-treatment *Varroa* was lower (average 0.58 mites per 100 bees across all treatment groups), oxalic acid alone did not provide effective *Varroa* control, but oxalic acid combined with Ecostep BC-12 did significantly reduce *Varroa* numbers. Mite drop data from year 1 indicated improved efficacy over the full 23-day treatment period for the oxalic acid with adjuvant treatment compared to glycerin control, but no difference between the oxalic acid with adjuvant and oxalic acid alone. However, over the first two days of treatment, increased mite drop was observed for the oxalic acid with adjuvant treatment relative to oxalic acid alone, indicating a more immediate impact of treatment when an adjuvant was included.

The decreased mite drop observed between the collection periods of days 7–9 and days 14–16 may indicate that extended-release oxalic acid treatments may become less effective over time (Table 3; S1 Text, Fig C). While studies have been performed on similar extended-release oxalic acid treatments, no published studies have addressed the persistence of oxalic acid in the treatments beyond taking measurements of *Varroa* levels in colonies. Yet, typical extended-release products for *Varroa* control are labeled for 42–56 days, indicating that residue testing on treatment strips would be useful in determining whether treatments are effective for a full 42- to 56-day treatment period. Comparisons of oxalic acid movement throughout the colony at multiple time points [80] would also be useful. Further testing is required to determine the optimal oxalic acid and adjuvant concentrations, and whether glycerin alone or a glycerin-water mixture should be used. Other off-label oxalic acid extended-release formulations have used a mixture of glycerin and water [78,79], which may change the activity of an adjuvant. While field experiments were performed in three geographically distinct apiaries, efficacy for many *Varroa* control miticides can vary with climate [79,81–84]. Further testing of this formulation should be performed in other climatic regions.

The increased mite drop observed over the first two days when an adjuvant was included may be the result of increased spreading throughout the colony. As all five adjuvants used in this study are listed by the manufacturer as surfactants, they may improve spreading on the honey bees or the *Varroa* themselves, thereby increasing the exposure of *Varroa* to the miticide. Increased spreading of the miticide throughout the colony and over the bee cuticle is critical in mediating exposure of *Varroa* to the miticide, as most *Varroa* on adult bees are located under the honey bee abdominal sternites [85]. In addition to improving spreading, adjuvants may also act through mechanisms that improve spiracular or cuticular penetration of the miticide in *Varroa*, as demonstrated in other arthropod pests [50,86–89]. It is also possible that adjuvants may have intrinsic toxicity to *Varroa*, as has been observed in spider mites [89–92], aphids [92–95], thrips [96], cockroaches [97], and mosquitoes [43,98]. However, the adjuvants used in this study were not tested on their own for toxicity to *Varroa*. Other adjuvants not used in this study are toxic to honey bees [45,99–104] and other bee species [105,106], so it is important to perform testing prior to including other adjuvants in candidate miticide formulations. The mechanism of action for adjuvant toxicity to arthropods is not well understood, but it is thought that adjuvants act similarly to insecticidal soaps [45,91,107,108], which can disrupt the arthropod cuticle, break down cell membranes, and reduce water surface tension to cause spiracular drowning [98,109].

Five adjuvant products, including two polyethylene/polypropylene glycol ethoxylates (Ecostep AE-13^®^ and BC-12^®^), two fatty acid ethoxylates (Ecostep CE-13^®^ and SE-11^®^), and one organo-silicone (Silwet L-7500 Copolymer^®^), were investigated in this study, but there are many other types of adjuvants that may improve efficacy of *Varroa* control applications, including other non-ionic surfactants, crop oils, seed oils, hydrocolloid polymers, or combinations [110]. Formulations of *Varroa* control products could take advantage of the spectrum of adjuvants to enhance new active ingredients for *Varroa* control [40,111]. Oxalic acid extended-release products utilizing adjuvants should be further developed and registered for use through the US EPA or the appropriate regulatory agency prior to use by beekeepers. There is a need to improve strategies for managing *Varroa*. Improving formulation chemistry is a critical tool to improve efficacy of chemical miticides that can be incorporated into an integrated pest management program [12,112]. This study is the first to demonstrate that adjuvants can improve the efficacy of miticide active ingredients against *Varroa*, which will provide better tools for beekeepers to control this devastating pest.

## Supporting information

S1 Text Supplemental Tables and Figures. **S1 Table A.** Bee mortality and *Varroa* mite drop efficacy for cage trials, expressed as a percentage, with range of standard error listed in parenthesis. The P-value of each treatment compared to the glycerin control and active ingredient control were determined via a pairwise Wilcoxon Rank-Sum Test using Benjamini-Hochberg post-hoc correction. Statistically significant differences (P < 0.05) are indicated with an asterisk (*).
**S1 Table B.** Year 1 and 2 ethanol wash data for each treatment, where rate is defined as *Varroa* infestation rate per 100 bees. The value listed in parenthesis for the initial and final rate mean indicates standard deviation; the range listed in parenthesis for the mean difference indicates standard error. The column “n” indicates the number of colonies used for analysis for each treatment. P-value within treatments was determined using a one-sided t-test on the difference within treatments and P-value between treatments and each of the controls was determined with an ANOVA with Tukey’s Post-Hoc test of the change in mite levels, where an asterisk (*) indicates a statistically significant test (P < 0.05).
**S1 Fig A.** Cage design for laboratory cage trials, with approximately 300 bees added to each cage.
**S1 Fig B.** The change in *Varroa* levels from alcohol washes for year 1 (A) and year 2 (B) field trials. Points indicate the post-treatment minus the pre-treatment *Varroa* mites per 100 bees for individual colonies. A statistically significant difference (P < 0.05) between post- and pre-treatment *Varroa* levels within treatments, determined using a GLMM, is indicated with an asterisk (*).
**S1 Fig C.** The daily mite drop for colonies in Year 1. Each point indicates the average daily mite drop over the 48-hour measurement period for an individual colony. Treatments were applied at day 0, immediately prior to sample collection.
(DOCX)

S2 Text R Code for Cage and Field Trial Data Analysis. (DOCX)

S3 Dataset Adjuvant Toxicity Testing Dataset. Adjuvant dose is listed as a percent composition by volume. Insecticide dose is listed as multiples of the application rate (X), where 1X Mustang Maxx is equivalent to 0.0186 percent composition by volume of formulation, or 0.00170 percent composition by weight of zeta-cypermethrin active ingredient.
(XLSX)

S4 Dataset Cage Trial Dataset. (XLSX)

S5 Dataset Year 1 Field Trial Dataset. (XLSX)

S6 Dataset Year 2 Field Trial Dataset. (XLSX)

## References

[1] Hristov P, Neov B, Shumkova R, Palova N. Significance of Apoidea as Main Pollinators. Ecological and Economic Impact and Implications for Human Nutrition. Diversity. 2020;12(7):280. doi: 10.3390/d12070280

[2] Sáez A, Aizen MA, Medici S, Viel M, Villalobos E, Negri P. Bees increase crop yield in an alleged pollinator-independent almond variety. Sci Rep. 2020;10(1):3177. doi: 10.1038/s41598-020-59995-0 32081891 PMC7035345

[3] U.S. Department of Agriculture. Nomadic commercial honey bee pollinators vital to some U.S. crops. 2014 [cited 28 Feb 2023]. Available from: http://www.ers.usda.gov/data-products/chart-gallery/gallery/chart-detail/?chartId=77714

[4] Calderone NW. Insect pollinated crops, insect pollinators and US agriculture: trend analysis of aggregate data for the period 1992-2009. PLoS One. 2012;7(5):e37235. doi: 10.1371/journal.pone.0037235 22629374 PMC3358326

[5] Khalifa SAM, Elshafiey EH, Shetaia AA, El-Wahed AAA, Algethami AF, Musharraf SG, et al. Overview of Bee Pollination and Its Economic Value for Crop Production. Insects. 2021;12(8):688. doi: 10.3390/insects12080688 34442255 PMC8396518

[6] Gallai N, Salles J-M, Settele J, Vaissière BE. Economic valuation of the vulnerability of world agriculture confronted with pollinator decline. Ecological Economics. 2009;68(3):810–21. doi: 10.1016/j.ecolecon.2008.06.014

[7] Giacobino A, Steinhauer N, Brunner S, Garcia-Andersen N, Aurell D, Rogers S, et al. Preliminary Results from the 2023-2024 US Beekeeping Survey: Colony Loss and Management. 2024. Available from: https://apiaryinspectors.org/US-beekeeping-survey

[8] Canadian Association of Professional Apiculturists (CAPA). Statement on honey bee wintering losses in Canada for 2024. 2024 [cited 5 Feb 2025]. Available from: https://capabees.com/capa-statement-on-honey-bees/.

[9] Aurell D, Bruckner S, Wilson M, Steinhauer N, Williams GR. A national survey of managed honey bee colony losses in the USA: Results from the Bee Informed Partnership for 2020–21 and 2021–22. Journal of Apicultural Research. 2023;63(1):1–14. doi: 10.1080/00218839.2023.2264601

[10] Steinhauer N, Wilson M, Aurell D, Bruckner S, Williams GR. United States Honey Bee Colony Losses 2022-2023: Preliminary Results from the Bee Informed Partnership. Bee Informed Partnership; 2023. Available from: https://beeinformed.org/wp-content/uploads/2023/06/BIP-2022-23-Loss-Abstract.pdf

[11] Gracia MJ, Moreno C, Ferrer M, Sanz A, Peribáñez MÁ, Estrada R. Field efficacy of acaricides against Varroa destructor. PLoS One. 2017;12(2):e0171633. doi: 10.1371/journal.pone.0171633 28158303 PMC5291502

[12] Jack CJ, Ellis JD. Integrated Pest Management Control of Varroa destructor (Acari: Varroidae), the Most Damaging Pest of (Apis mellifera L. (Hymenoptera: Apidae)) Colonies. J Insect Sci. 2021;21(5):6. doi: 10.1093/jisesa/ieab058 34536080 PMC8449538

[13] Elzen PJ, Baxter JR, Spivak M, Wilson WT. Control ofVarroa jacobsoniOud. resistant to fluvalinate and amitraz using coumaphos. Apidologie. 2000;31(3):437–41. doi: 10.1051/apido:2000134

[14] Kamler M, Nesvorna M, Stara J, Erban T, Hubert J. Comparison of tau-fluvalinate, acrinathrin, and amitraz effects on susceptible and resistant populations of Varroa destructor in a vial test. Exp Appl Acarol. 2016;69(1):1–9. doi: 10.1007/s10493-016-0023-8 26910521

[15] Maggi MD, Ruffinengo SR, Negri P, Eguaras MJ. Resistance phenomena to amitraz from populations of the ectoparasitic mite Varroa destructor of Argentina. Parasitol Res. 2010;107(5):1189–92. doi: 10.1007/s00436-010-1986-8 20668878

[16] Rinkevich FD, Moreno-Martí S, Hernández-Rodríguez CS, González-Cabrera J. Confirmation of the Y215H mutation in the β2 -octopamine receptor in Varroa destructor is associated with contemporary cases of amitraz resistance in the United States. Pest Manag Sci. 2023;79(8):2840–5. doi: 10.1002/ps.7461 36947601

[17] Rinkevich FD. Detection of amitraz resistance and reduced treatment efficacy in the Varroa Mite, Varroa destructor, within commercial beekeeping operations. PLoS One. 2020;15(1):e0227264. doi: 10.1371/journal.pone.0227264 31951619 PMC6968863

[18] Rodríguez-Dehaibes SR, Otero-Colina G, Sedas VP, Jiménez JAV. Resistance to amitraz and flumethrin inVarroa destructorpopulations from Veracruz, Mexico. Journal of Apicultural Research. 2005;44(3):124–5. doi: 10.1080/00218839.2005.11101162

[19] Maggi MD, Ruffinengo SR, Damiani N, Sardella NH, Eguaras MJ. First detection of Varroa destructor resistance to coumaphos in Argentina. Exp Appl Acarol. 2009;47(4):317–20. doi: 10.1007/s10493-008-9216-0 19009360

[20] Medici SK, Maggi MD, Sarlo EG, Ruffinengo S, Marioli JM, Eguaras MJ. The presence of synthetic acaricides in beeswax and its influence on the development of resistance in Varroa destructor. Journal of Apicultural Research. 2015;54(3):267–74. doi: 10.1080/00218839.2016.1145407

[21] Pettis JS. A scientific note on Varroa destructor resistance to coumaphos in the United States. Apidologie. 2004;35(1):91–2. doi: 10.1051/apido:2003060

[22] Spreafico M, Eördegh FR, Bernardinelli I, Colombo M. First detection of strains of Varroa destructor resistant to coumaphos. Results of laboratory tests and field trials. Apidologie. 2001;32(1):49–55. doi: 10.1051/apido:2001110

[23] González-Cabrera J, Bumann H, Rodríguez-Vargas S, Kennedy PJ, Krieger K, Altreuther G, et al. A single mutation is driving resistance to pyrethroids in European populations of the parasitic mite, Varroa destructor. J Pest Sci. 2018;91(3):1137–44. doi: 10.1007/s10340-018-0968-y

[24] Lodesani M, Colombo M, Spreafico M. Ineffectiveness of Apistan® treatment against the mite Varroa jacobsoni Oud in several districts of Lombardy (Italy). Apidologie. 1995;26(1):67–72. doi: 10.1051/apido:19950109

[25] Miozes-Koch R, Slabezki Y, Efrat H, Kalev H, Kamer Y, , et al. First detection in Israel of fluvalinate resistance in the varroa mite using bioassay and biochemical methods. Exp Appl Acarol. 2000;24(1):35–43. doi: 10.1023/a:1006379114942 10823355

[26] Thompson HM, Brown MA, Ball RF, Bew MH. First report of Varroa destructor resistance to pyrethroids in the UK. Apidologie. 2002;33(4):357–66. doi: 10.1051/apido:2002027

[27] Popov E, Melnik V, Matchinev A. Application of oxalic acid in varroatosis. In: Proc XXXII Int Congr Apimondia. Apimondia Publ House. 1989. 149.

[28] Maggi M, Damiani N, Ruffinengo S, Brasesco M, Szawarski N, Mitton G. The susceptibility of Varroa destructor against oxalic acid: A study case. Bull Insectol. 2016;70:39–44.

[29] Howis M, Chorbiński P, Nowakowski P. Physical damage to the chitin plate and position of Varroa destructor on hive bottoms after use of different varroacidal treatments. Med Weter. 2012;68:607–11.

[30] Gregorc A, Adamczyk J, Kapun S, Planinc I. Integrated varroa control in honey bee (Apis mellifera carnica) colonies with or without brood. Journal of Apicultural Research. 2016;55(3):253–8. doi: 10.1080/00218839.2016.1222700

[31] Gregorc A, Planinc I. Acaricidal effect of oxalic acid in honeybee (apis mellifera) colonies. Apidologie. 2001;32(4):333–40. doi: 10.1051/apido:2001133

[32] Oliver R. Instructions for extended-release oxalic acid. In: Scientific Beekeeping [Internet]. 4 Jul 2022 [cited 6 Aug 2024]. Available from: https://scientificbeekeeping.com/instructions-for-extended-release-oxalic-acid/

[33] Gashout HA, Guzmán-Novoa E. Acute toxicity of essential oils and other natural compounds to the parasitic mite, Varroa destructor, and to larval and adult worker honey bees (Apis mellifera L.). Journal of Apicultural Research. 2009;48(4):263–9. doi: 10.3896/ibra.1.48.4.06

[34] Goswami V, Khan M. Management of varroa mite, Varroa destructor by essential oil and formic acid in Apis mellifera Linn. colonies. J Nat Prod. 2013;6:206–10.

[35] Mahmood R, Asad S, Raja S, ul Moshin A, Wagchoure ES, Sarwar G. Control of varroa destructor (acari: varroidae) in apis mellifera (hymenoptera: apidae) by using plant oils and extract. Pak J Zool. 2014;46.

[36] Su X, Zheng H, Fei Z, Hu F. Effectiveness of herbal essential oils as fumigants to control Varroa destructor in laboratory assays. Chin J Appl Entomol. 2012;5:1189–95.

[37] Lewis KA, Tzilivakis J, Warner DJ, Green A. An international database for pesticide risk assessments and management. Human and Ecological Risk Assessment: An International Journal. 2016;22(4):1050–64. doi: 10.1080/10807039.2015.1133242

[38] Li L, Lin Z-G, Wang S, Su X-L, Gong H-R, Li H-L, et al. The effects of clove oil on the enzyme activity of Varroa destructor Anderson and Trueman (Arachnida: Acari: Varroidae). Saudi J Biol Sci. 2017;24(5):996–1000. doi: 10.1016/j.sjbs.2017.01.052 28663694 PMC5478387

[39] IRAC - Insecticide Resistance Action Committee. Mode of Action Classification | Insecticide Resistance Management. In: IRAC [Internet]. 2024 [cited 26 Mar 2024]. Available: https://irac-online.org/mode-of-action/classification-online/.

[40] Bahreini R, Nasr M, Docherty C, Muirhead S, de Herdt O, Feindel D. Miticidal activity of fenazaquin and fenpyroximate against Varroa destructor, an ectoparasite of Apis mellifera. Pest Manag Sci. 2022;78(4):1686–97. doi: 10.1002/ps.6788 34994089 PMC9303763

[41] US EPA O. EPA-registered Pesticide Products Approved for Use Against Varroa Mites in Bee Hives. 2016 [cited 3 Feb 2025]. Available from: https://www.epa.gov/pollinator-protection/epa-registered-pesticide-products-approved-use-against-varroa-mites-bee-hives

[42] Young BG, Matthews JL, Whitford F. Compendium of herbicide adjuvants. 2016. Available from: http://siu-weeds.com/adjuvants/index-adj.html

[43] Shannon B, Jeon H, Johnson RM. Review: the risks of spray adjuvants to honey bees. J Insect Sci. 2023;23(6):20. doi: 10.1093/jisesa/iead100 38055940 PMC10699877

[44] US EPA. Inert Ingredients Overview and Guidance. 5 Mar 2013 [cited 14 Jun 2023]. Available from: https://www.epa.gov/pesticide-registration/inert-ingredients-overview-and-guidance.

[45] Shannon B, Walker E, Johnson RM. Toxicity of spray adjuvants and tank mix combinations used in almond orchards to adult honey bees (Apis mellifera). J Econ Entomol. 2023;116(5):1467–80. doi: 10.1093/jee/toad161 37656894 PMC10564267

[46] MarketsandMarkets. Agricultural Adjuvants Market Industry Analysis | Types, Advantages, and Forecast. 2023. Available from: https://www.marketsandmarkets.com/Market-Reports/adjuvant-market-1240.html.

[47] US EPA O. Advisory on the Applicability of FIFRA and FFDCA for Substances used to Control Varroa Mites in Beehives. 5 Jan 2024 [cited 22 Jan 2024]. Available from: https://www.epa.gov/pollinator-protection/advisory-applicability-fifra-and-ffdca-substances-used-control-varroa-mites

[48] Potter C. An improved laboratory apparatus for applying direct sprays and surface films, with data on the electrostatic charge on atomized spray fluids. Annals of Applied Biology. 1952;39(1):1–28. doi: 10.1111/j.1744-7348.1952.tb00993.x

[49] Zhu YC, Adamczyk J, Rinderer T, Yao J, Danka R, Luttrell R, et al. Spray Toxicity and Risk Potential of 42 Commonly Used Formulations of Row Crop Pesticides to Adult Honey Bees (Hymenoptera: Apidae). J Econ Entomol. 2015;108(6):2640–7. doi: 10.1093/jee/tov269 26352753

[50] Fanning PD, VanWoerkom A, Wise JC, Isaacs R. Assessment of a commercial spider venom peptide against spotted-wing Drosophila and interaction with adjuvants. J Pest Sci. 2018;91(4):1279–90. doi: 10.1007/s10340-018-1016-7

[51] Walker EK, Brock GN, Arvidson RS, Johnson RM. Acute Toxicity of Fungicide-Insecticide-Adjuvant Combinations Applied to Almonds During Bloom on Adult Honey Bees. Environ Toxicol Chem. 2022;41(4):1042–53. doi: 10.1002/etc.5297 35060643 PMC9313819

[52] Ritz C, Baty F, Streibig JC, Gerhard D. Dose-Response Analysis Using R. PLoS One. 2015;10(12):e0146021. doi: 10.1371/journal.pone.0146021 26717316 PMC4696819

[53] Chattaway FD. XX.—Interaction of glycerol and oxalic acid. J Chem Soc, Trans. 1914;105(0):151–6. doi: 10.1039/ct9140500151

[54] Alksnis AF, Gruziń IV, Surna YaA. Synthesis of oligoesters from oxalic acid and glycerol. J Polym Sci Polym Chem Ed. 1976;14(11):2631–8. doi: 10.1002/pol.1976.170141104

[55] Oliver R. 2022 Extended-Release Oxalic Update: Part 1. In: Scientific Beekeeping [Internet]. 22 Apr 2022 [cited 28 Oct 2022]. Available from: https://scientificbeekeeping.com/7701-2/.

[56] Vermont Agency of Agriculture Food and Markets. 2ee Recommendations for the use of for Api-Bioxal (EPA Reg. No. 91266-1-73291)-Oxalic Acid. 2022. Available from: https://agriculture.vermont.gov/sites/agriculture/files/Oxalic%202ee.pdf

[57] Imdorf A, Buehlmann G, Gerig L, Kilchenmann V, Wille H. Überprüfung Der Schätzmethode Zur Ermittlung Der Brutfläche Und Der Anzahl Arbeiterinnen In Freifliegenden Bienenvölkern. Apidologie. 1987;18(2):137–46. doi: 10.1051/apido:19870204

[58] Dainat B, Dietemann V, Imdorf A, Charrière J-D. A scientific note on the ‘Liebefeld Method’ to estimate honey bee colony strength: its history, use, and translation. Apidologie. 2020;51(3):422–7. doi: 10.1007/s13592-019-00728-2

[59] Bahreini R, Nasr M, Docherty C, Feindel D, Muirhead S, de Herdt O. New bioassay cage methodology for in vitro studies on Varroa destructor and Apis mellifera. PLoS One. 2021;16(4):e0250594. doi: 10.1371/journal.pone.0250594 33901245 PMC8075223

[60] Wahl O, Ulm K. Influence of pollen feeding and physiological condition on pesticide sensitivity of the honey bee Apis mellifera carnica. Oecologia. 1983;59(1):106–28. doi: 10.1007/BF00388082 25024157

[61] Mondet F, Goodwin M, Mercer A. Age-related changes in the behavioural response of honeybees to Apiguard®, a thymol-based treatment used to control the mite Varroa destructor. J Comp Physiol A Neuroethol Sens Neural Behav Physiol. 2011;197(11):1055–62. doi: 10.1007/s00359-011-0666-1 21761187

[62] Baines D, Wilton E, Pawluk A, de Gorter M, Chomistek N. Neonicotinoids act like endocrine disrupting chemicals in newly-emerged bees and winter bees. Sci Rep. 2017;7(1):10979. doi: 10.1038/s41598-017-10489-6 28887455 PMC5591280

[63] Boecking O, Genersch E. Varroosis – the Ongoing Crisis in Bee Keeping. J Verbr Lebensm. 2008;3(2):221–8. doi: 10.1007/s00003-008-0331-y

[64] Rosenkranz P, Aumeier P, Ziegelmann B. Biology and control of Varroa destructor. J Invertebr Pathol. 2010;103 Suppl 1:S96-119. doi: 10.1016/j.jip.2009.07.016 19909970

[65] Traynor KS, Mondet F, de Miranda JR, Techer M, Kowallik V, Oddie MAY, et al. Varroa destructor: A Complex Parasite, Crippling Honey Bees Worldwide. Trends Parasitol. 2020;36(7):592–606. doi: 10.1016/j.pt.2020.04.004 32456963

[66] Fries I, Aarhus A, Hansen H, Korpela S. Comparison of diagnostic methods for detection of low infestation levels ofVarroa jacobsoni in honey-bee (Apis mellifera) colonies. Exp Appl Acarol. 1991;10(3–4):279–87. doi: 10.1007/bf01198656

[67] Dietemann V, Nazzi F, Martin SJ, Anderson DL, Locke B, Delaplane KS, et al. Standard methods for varroa research. Journal of Apicultural Research. 2013;52(1):1–54. doi: 10.3896/ibra.1.52.1.09 PMC3816652 24198438

[68] Cox DR. The Regression Analysis of Binary Sequences. Journal of the Royal Statistical Society Series B: Statistical Methodology. 1958;20(2):215–32. doi: 10.1111/j.2517-6161.1958.tb00292.x

[69] Keselman HJ, Rogan JC. The Tukey multiple comparison test: 1953–1976. Psychological Bulletin. 1977;84(5):1050–6. doi: 10.1037/0033-2909.84.5.1050

[70] McGregor SE, Rowe JB. Honey bee colony quality for alfalfa pollination. Am Bee J. 1979;119:700–3, 761–5.

[71] Nasr ME, Thorp RW, Tyler TL, Briggs DL. Estimating Honey Bee (Hymenoptera: Apidae) Colony Strength by a Simple Method: Measuring Cluster Size. Journal of Economic Entomology. 1990;83(3):748–54. doi: 10.1093/jee/83.3.748

[72] Lin C-H, Sponsler DB, Richardson RT, Watters HD, Glinski DA, Henderson WM, et al. Honey Bees and Neonicotinoid-Treated Corn Seed: Contamination, Exposure, and Effects. Environ Toxicol Chem. 2021;40(4):1212–21. doi: 10.1002/etc.4957 33289922 PMC8048971

[73] Bolker BM, Brooks ME, Clark CJ, Geange SW, Poulsen JR, Stevens MHH, et al. Generalized linear mixed models: a practical guide for ecology and evolution. Trends Ecol Evol. 2009;24(3):127–35. doi: 10.1016/j.tree.2008.10.008 19185386

[74] Davis RA, Wu R. A negative binomial model for time series of counts. Biometrika. 2009;96(3):735–49. doi: 10.1093/biomet/asp029

[75] Maggi M, Tourn E, Negri P, Szawarski N, Marconi A, Gallez L, et al. A new formulation of oxalic acid for Varroa destructor control applied in Apis mellifera colonies in the presence of brood. Apidologie. 2015;47(4):596–605. doi: 10.1007/s13592-015-0405-7

[76] Rodríguez Dehaibes SR, Meroi Arcerito FR, Chávez-Hernández E, Luna-Olivares G, Marcangeli J, Eguaras M, et al. Control of Varroa destructor development in Africanized Apis mellifera honeybees using Aluen Cap (oxalic acid formulation). International Journal of Acarology. 2020;46(6):405–8. doi: 10.1080/01647954.2020.1806923

[77] Sabahi Q, Morfin N, Nehzati-Paghaleh G, Guzman-Novoa E. Detection and replication of deformed wing virus and black queen cell virus in parasitic mites, Varroa destructor, from Iranian honey bee (Apis mellifera) colonies. Journal of Apicultural Research. 2019;59(2):211–7. doi: 10.1080/00218839.2019.1686576

[78] Kanelis D, Tananaki C, Liolios V, Rodopoulou M. Evaluation of oxalic acid with glycerin efficacy against Varroa destructor (Varroidae): a four year assay. Journal of Apicultural Research. 2023;63(5):847–55. doi: 10.1080/00218839.2023.2169368

[79] Bartlett LJ, Baker C, Bruckner S, Delaplane KS, Hackmeyer EJ, Phankaew C, et al. No evidence to support the use of glycerol-oxalic acid mixtures delivered via paper towel for controlling Varroa destructor (Mesostigmata: Varroidae) mites in the Southeast United States. J Insect Sci. 2023;23(6):18. doi: 10.1093/jisesa/iead097 38055939 PMC10699872

[80] Oliver R. Oxalic crystals on bees after vaporization. In: Scientific Beekeeping [Internet]. 25 Mar 2021 [cited 24 Jan 2024]. Available: https://scientificbeekeeping.com/oxalic-crystals-on-bees-after-vaporization/

[81] Bacandritsos N, Papanastasiou I, Saitanis C, Nanetti A, Roinioti E. Efficacy of repeated trickle applications of oxalic acid in syrup for varroosis control in Apis mellifera: influence of meteorological conditions and presence of brood. Vet Parasitol. 2007;148(2):174–8. doi: 10.1016/j.vetpar.2007.06.001 17624673

[82] Gregorc A, Planinc I. Use of Thymol Formulations, Amitraz, and Oxalic Acid for the Control of the Varroa Mite in Honey Bee (Apis mellifera carnica) Colonies. Journal of Apicultural Science. 2012;56(2):61–9. doi: 10.2478/v10289-012-0024-8

[83] Patricia A, Rafael R, Alejandra O, Macarena F, Daniel R, Fanny N. Effect of ambient temperature and humidity conditions on the efficacy of organic treatments against Varroa destructor in different climatic zones of Chile. J Agric Sci Technol A. 2013;3:474.

[84] Gregorc A, Alburaki M, Werle C, Knight PR, Adamczyk J. Brood removal or queen caging combined with oxalic acid treatment to control varroa mites (Varroa destructor) in honey bee colonies (Apis mellifera). Apidologie. 2017;48(6):821–32. doi: 10.1007/s13592-017-0526-2

[85] Ramsey SD, Ochoa R, Bauchan G, Gulbronson C, Mowery JD, Cohen A, et al. Varroa destructor feeds primarily on honey bee fat body tissue and not hemolymph. Proc Natl Acad Sci U S A. 2019;116(5):1792–801. doi: 10.1073/pnas.1818371116 30647116 PMC6358713

[86] HURST H. Permeability of Insect Cuticle. Nature. 1940;145(3673):462–3. doi: 10.1038/145462b0

[87] Olson WP, O’Brien RD. The relation between physical properties and penetration of solutes into the cockroach cuticle. Journal of Insect Physiology. 1963;9(6):777–86. doi: 10.1016/0022-1910(63)90037-4

[88] Lewis CT. The Penetration of Cuticle by Insecticides. In: Miller TA, Editor. Cuticle Techniques in Arthropods. New York, NY: Springer; 1980. pp. 367–400. doi: 10.1007/978-1-4612-6076-9_10

[89] Dentener PR, Peetz SM. Postharvest control of diapausing two-spotted spider mite *Tetranychus urticae* Koch on fruit. I Comparison of insecticidal soaps and spray adjuvants. pnzppc. 1992;45:116–20. doi: 10.30843/nzpp.1992.45.11206

[90] Searle GG, Penman DR, Chapman RB. The toxicity of herbicides to the gorse spider mite *Tetranychus lintearius*. pnzwpcc. 1990;43:178–81. doi: 10.30843/nzpp.1990.43.10896

[91] Cowles RS, Cowles EA, McDermott AM, Ramoutar D. “Inert” formulation ingredients with activity: toxicity of trisiloxane surfactant solutions to twospotted spider mites (Acari: Tetranychidae). J Econ Entomol. 2000;93(2):180–8. doi: 10.1603/0022-0493-93.2.180 10826161

[92] Cloyd R. Do surfactants kill insects and mites?. GMPro. 2005;25:62.

[93] Wolfenbarger DA. Oils and Surfactants Alone, and Insecticide-Oil Combinations for Aphid Control on Turnips and Cabbage1. Journal of Economic Entomology. 1964;57(4):571–4. doi: 10.1093/jee/57.4.571

[94] Radwan HSA, Mesbah HA, Abdel‐Fattah MS, El‐Mohymen MRA, Hassan NA. The effect of various adjuvants on the insecticidal activity of diflubenzuron against the cabbage aphid, Brevicoryne brassicae (L.). Zeitschrift für Angewandte Entomologie. 1982;94(1–5):420–3. doi: 10.1111/j.1439-0418.1982.tb02588.x

[95] Acheampong S, Stark JD. Effects of the agricultural adjuvant Sylgard 309 and the insecticide pymetrozine on demographic parameters of the aphid parasitoid, Diaeretiella rapae. Biological Control. 2004;31(2):133–7. doi: 10.1016/j.biocontrol.2004.03.010

[96] Tang L-D, Guo L-H, Ali A, Desneux N, Zang L-S. Synergism of Adjuvants Mixed With Spinetoram for the Management of Bean Flower Thrips, Megalurothrips usitatus (Thysanoptera: Thripidae) in Cowpeas. J Econ Entomol. 2022;115(6):2013–9. doi: 10.1093/jee/toac149 36178344

[97] Sims SR, Appel AG. Linear Alcohol Ethoxylates: Insecticidal and Synergistic Effects on German Cockroaches (Blattodea: Blattellidae) and Other Insects. Journal of Economic Entomology. 2007;100(3):871–9. doi: 10.1093/jee/100.3.871 17598550

[98] Ware GW, Whitacre DM. The pesticide book. 6th ed. ed. MeisterPro Information Resources. 2004.

[99] Goodwin RM, McBrydie HM. Effect of surfactants on honey bees. NZPP. 2000;53:230–4. doi: 10.30843/nzpp.2000.53.3694

[100] Ciarlo TJ, Mullin CA, Frazier JL, Schmehl DR. Learning impairment in honey bees caused by agricultural spray adjuvants. PLoS One. 2012;7(7):e40848. doi: 10.1371/journal.pone.0040848 22815841 PMC3397935

[101] Zhu W, Schmehl DR, Mullin CA, Frazier JL. Four common pesticides, their mixtures and a formulation solvent in the hive environment have high oral toxicity to honey bee larvae. PLoS One. 2014;9(1):e77547. doi: 10.1371/journal.pone.0077547 24416121 PMC3885384

[102] Mullin CA, Chen J, Fine JD, Frazier MT, Frazier JL. The formulation makes the honey bee poison. Pestic Biochem Physiol. 2015;120:27–35. doi: 10.1016/j.pestbp.2014.12.026 25987217

[103] Fine JD, Cox-Foster DL, Mullin CA. An Inert Pesticide Adjuvant Synergizes Viral Pathogenicity and Mortality in Honey Bee Larvae. Sci Rep. 2017;7:40499. doi: 10.1038/srep40499 28091574 PMC5238421

[104] Ricke DF, Lin C-H, Johnson RM. Pollen Treated with a Combination of Agrochemicals Commonly Applied During Almond Bloom Reduces the Emergence Rate and Longevity of Honey Bee (Hymenoptera: Apidae) Queens. J Insect Sci. 2021;21(6):5. doi: 10.1093/jisesa/ieab074 34723328 PMC8559157

[105] Artz DR, Pitts-Singer TL. Effects of Fungicide and Adjuvant Sprays on Nesting Behavior in Two Managed Solitary Bees, Osmia lignaria and Megachile rotundata. PLoS One. 2015;10(8):e0135688. doi: 10.1371/journal.pone.0135688 26274401 PMC4537283

[106] Straw EA, Brown MJF. Co-formulant in a commercial fungicide product causes lethal and sub-lethal effects in bumble bees. Sci Rep. 2021;11(1):21653. doi: 10.1038/s41598-021-00919-x 34741036 PMC8571393

[107] Cirelli AF, Ojeda C, Castro MJL, Salgot M. Surfactants in Sludge-Amended Agricultural Soils: A Review. In: Lichtfouse E, Editor. Organic Farming, Pest Control and Remediation of Soil Pollutants: Organic farming, pest control and remediation of soil pollutants. Dordrecht: Springer Netherlands; 2010. pp. 227–51. doi: 10.1007/978-1-4020-9654-9_12

[108] Castro MJL, Ojeda C, Cirelli AF. Advances in surfactants for agrochemicals. Environ Chem Lett. 2013;12(1):85–95. doi: 10.1007/s10311-013-0432-4

[109] Yu SJ. The toxicology and biochemistry of insecticides. CRC Press. 2008.

[110] Abbott JR, Zhu H, Jeon H. Retention and Spread Capability of Impacted Droplets with Surfactant and Hydrocolloid Based Adjuvants. Transactions of the ASABE. 2021;64(6):1883–94. doi: 10.13031/trans.14461

[111] Jack CJ, Kleckner K, Demares F, Rault LC, Anderson TD, Carlier PR, et al. Testing new compounds for efficacy against Varroa destructor and safety to honey bees (Apis mellifera). Pest Manag Sci. 2022;78(1):159–65. doi: 10.1002/ps.6617 34464499

[112] Vilarem C, Piou V, Vogelweith F, Vétillard A. Varroa destructor from the Laboratory to the Field: Control, Biocontrol and IPM Perspectives-A Review. Insects. 2021;12(9):800. doi: 10.3390/insects12090800 34564240 PMC8465918

