# Supplementary material for: Adjuvants to improve efficacy of miticides in managed honey bee (Apis mellifera) colonies to control Varroa destructor
Source: PLoS One. 2025 Jun 17;20(6):e0320037. doi: 10.1371/journal.pone.0320037 (PMC12173398; doi:10.1371/journal.pone.0320037)
Supplement: S1 Text — S1 Table A. Bee mortality and Varroa mite drop efficacy for cage trials, expressed as a percentage, with range of standard error listed in parenthesis. The P-value of each treatment compared to the glycerin control and active ingredient control were determined via a pairwise Wilcoxon Rank-Sum Test using Benjamini-Hochberg post-hoc correction. Statistically significant differences (P < 0.05) are indicated with an asterisk (*). S1 Table B. Year 1 and 2 ethanol wash data for each treatment, where rate is defined as Varroa infestation rate per 100 bees. The value listed in parenthesis for the initial and final rate mean indicates standard deviation; the range listed in parenthesis for the mean difference indicates standard error. The column “n” indicates the number of colonies used for analysis for each treatment. P-value within treatments was determined using a one-sided t-test on the difference within treatments and P-value between treatments and each of the controls was determined with an ANOVA with Tukey’s Post-Hoc test of the change in mite levels, where an asterisk (*) indicates a statistically significant test (P < 0.05). S1 Fig A. Cage design for laboratory cage trials, with approximately 300 bees added to each cage. S1 Fig B. The change in Varroa levels from alcohol washes for year 1 (A) and year 2 (B) field trials. Points indicate the post-treatment minus the pre-treatment Varroa mites per 100 bees for individual colonies. A statistically significant difference (P < 0.05) between post- and pre-treatment Varroa levels within treatments, determined using a GLMM, is indicated with an asterisk (*). S1 Fig C. The daily mite drop for colonies in Year 1. Each point indicates the average daily mite drop over the 48-hour measurement period for an individual colony. Treatments were applied at day 0, immediately prior to sample collection. (DOCX) [file pone.0320037.s001.docx]

# S1. Text. Supplemental Tables and Figures.

**S1 Table A.** Bee mortality and *Varroa* mite drop efficacy for cage trials, expressed as a percentage, with range of standard error listed in parenthesis. The P-value of each treatment compared to the glycerin control and active ingredient control were determined via logistic regression followed by Tukey HSD post-hoc test. Statistically significant differences (P < 0.05) are indicated with an asterisk (*).

| Treatment | n | 24-hour bee mortality (%) | Bee mortality P-value to glycerin control | 24-hour efficacy (%) | Efficacy P-value to glycerin control | Efficacy P-value to active ingredient control |
| --- | --- | --- | --- | --- | --- | --- |
| Clove Oil Solvent Control | 6 | 0.6  (0.5) | – | 26.3  (12.9) | – | – |
| Clove Oil Active Ingredient Control | 3 | 0.7  (0.6) | 0.907 | 27.3  (17.0) | 0.815 | – |
| Ecostep AE-13® | 6 | 0.7  (0.8) | 1.000 | 69.9  (10.6) | < 0.001* | < 0.001* |
| Ecostep BC-12® | 6 | 1.3  (1.8) | 0.050* | 64.7  (27.0) | < 0.001* | < 0.001* |
| Ecostep SE-11® | 3 | 0.9  (0.7) | 0.626 | 46.8  (17.3) | 0.0129* | 0.374 |
| Silwet L-7500 Copolymer® | 7 | 1.4  (1.4) | 0.112 | 59.5  (11.1) | < 0.001* | 0.015* |
| Fenpyroximate Solvent Control | 5 | 0.2  (0.2) | – | 28.9  (5.5) | - | - |
| Fenpyroximate Active Ingredient Control | 5 | 0.6  (1.2) | 0.251 | 51.6  (21.0) | 0.0231* | - |
| Ecostep BC-12® | 6 | 3.0  (3.1) | < 0.001* | 75.1  (26.7) | < 0.001* | < 0.001* |
| Ecostep CE-13® | 5 | 4.2  (3.6) | < 0.001* | 64.0  (31.9) | < 0.001* | < 0.001* |
| Ecostep SE-11® | 5 | 4.3  (6.7) | < 0.001* | 66.8  (34.4) | < 0.001* | < 0.001* |
| Silwet L-7500 Copolymer® | 5 | 1.9  (2.0) | 0.001* | 62.9  (13.0) | < 0.001* | 0.0326* |
| Oxalic Acid Solvent Control | 5 | 0.4  (0.4) | – | 27.5  (10.5) | – | – |
| Oxalic Acid Active Ingredient Control | 5 | 0.1  (0.2) | 0.529 | 75.9  (17.1) | < 0.001* | – |
| Ecostep BC-12® | 5 | 0.7  (1.0) | 0.893 | 96.4  (5.0) | < 0.001* | 0.012* |
| Ecostep CE-13® | 5 | 0.5  (0.4) | 0.968 | 86.5  (14.1) | < 0.001* | 0.632 |
| Ecostep SE-11® | 5 | 0.7  (0.4) | 0.733 | 88.3  (5.3) | < 0.001* | 0.067 |
| Silwet L-7500 Copolymer® | 5 | 0.5  (0.8) | 0.987 | 84.6  (11.4) | < 0.001* | 0.634 |

**S1 Table B.** Year 1 and 2 ethanol wash data for each treatment, where rate is defined as *Varroa* infestation rate per 100 bees. The value listed in parenthesis for the initial and final rate mean indicates standard deviation; the range listed in parenthesis for the mean difference indicates standard error. The column “n” indicates the number of colonies used for analysis for each treatment. P-value compares post- and pre-treatment *Varroa* levels within treatments using a GLMM, where an asterisk (*) indicates a statistically significant difference (P < 0.05).

| Treatment | n | Initial Rate Mean | Final Rate  Mean | Mean Difference | P-value |
| --- | --- | --- | --- | --- | --- |
| Glycerin control (Year 1) | 5 | 12.4  (7.1) | 20.8  (16.3) | 8.4  (1.7 – 15.1) | 0.229 |
| Oxalic Acid Control (Year 1) | 7 | 12.1  (7.6) | 7.6  (9.6) | -4.3  (-8.7 – 0.1) | 0.115 |
| Oxalic Acid Plus Adjuvant (Year 1) | 6 | 10.9  (7.1) | 7.8  (7.5) | -3.1  (-4.1 – -2.1) | 0.248 |
| Glycerin control (Year 2) | 9 | 0.8  (0.8) | 2.1  (0.9) | 1.3  (0.9 – 1.7) | < 0.0001* |
| Oxalic Acid Control (Year 2) | 9 | 0.4  (0.5) | 1.0  (0.5) | 0.7  (0.5 – 0.8) | < 0.0001* |
| Oxalic Acid Plus Adjuvant (Year 2) | 9 | 0.6  (0.7) | 0.4  (0.4) | -0.2  (-0.5 – 0.2) | < 0.0001* |


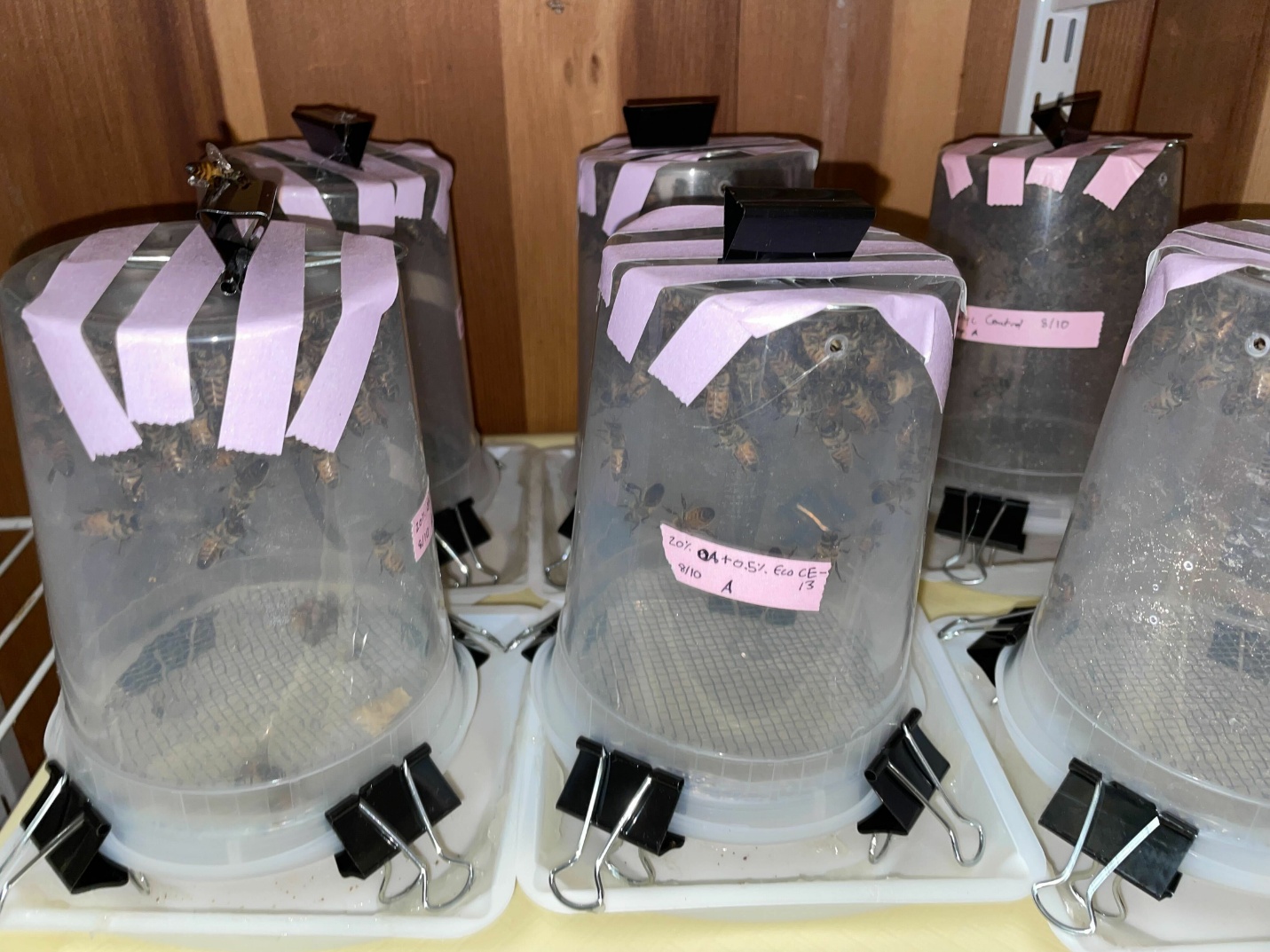


**S1 Fig A.** Cage design for laboratory cage trials, with approximately 300 bees added to each cage.





**S1 Fig B.** The change in *Varroa* levels from alcohol washes for year 1 (A) and year 2 (B) field trials. Points indicate the post-treatment minus the pre-treatment *Varroa* mites per 100 bees for individual colonies. A statistically significant difference (P < 0.05) between post- and pre-treatment *Varroa* levels within treatments, determined using a GLMM, is indicated with an asterisk (*).


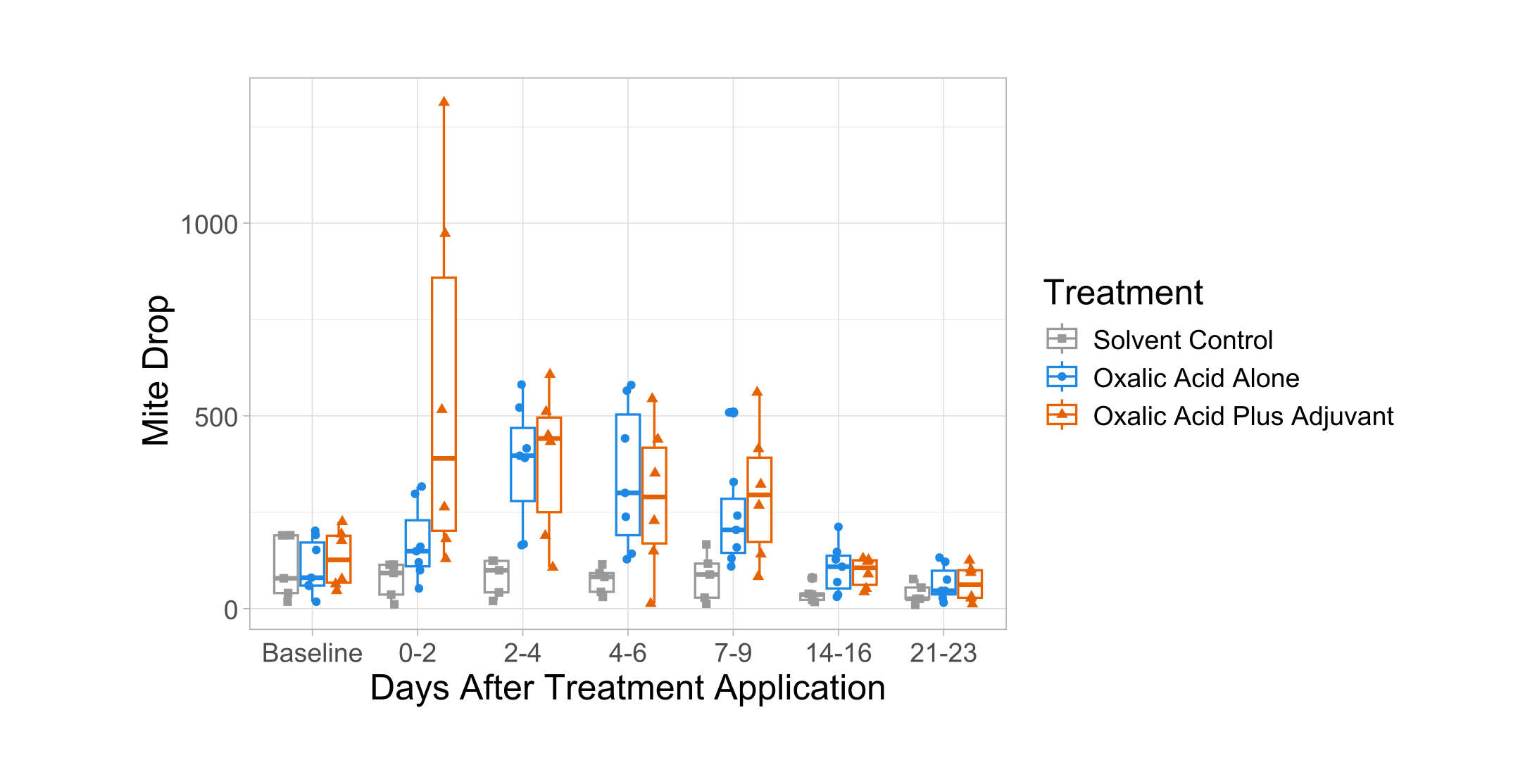


**S1 Fig C.** The daily mite drop for colonies in Year 1. Each point indicates the average daily mite drop over the 48-hour measurement period for an individual colony. Treatments were applied at day 0, immediately prior to sample collection.
