# Supplementary material for: Adjuvants to improve efficacy of miticides in managed honey bee (Apis mellifera) colonies to control Varroa destructor
Source: PLoS One. 2025 Jun 17;20(6):e0320037. doi: 10.1371/journal.pone.0320037 (PMC12173398; doi:10.1371/journal.pone.0320037)
Supplement: S2 Text — (DOCX) [file pone.0320037.s002.docx]

**S2 Text. R Code for Cage and Field Trial Data Analysis.**

library(readxl)

library(tidyr)

library(nlme)

library(dplyr)

library(contrast)

library(car)

library(emmeans)

library(multcomp)

library(ggplot2)

library(reshape2)

library(MASS)

library(tidyverse)

library(lme4)

# Set working directory

setwd("D:/consulting/honey bee")

# Cage Trials---------------------------------

# Load the data

data_cage <- S4_Dataset

# change column names for ease of use in R

colnames(data_cage)[9] <- "bees.dead"

colnames(data_cage)[10] <- "varroa.dead"

colnames(data_cage)[12] <- "varroa.wash"

colnames(data_cage)[15] <- "total.bees"

data_cage$bees.alive <- (data_cage$total.bees - data_cage$bees.dead)

data_cage$bees.alive <- round(data_cage$bees.alive,0)

# filter data subsets

data_cage_clove <- data_cage[1:31,] %>%

mutate(PFA = case_when(AI == "Control" ~ "GlyControl", .default = PFA))

data_cage_fen <- data_cage[33:63,] %>%

mutate(PFA = case_when(AI == "Control" ~ "GlyControl", .default = PFA))

data_cage_oxalic <- data_cage[65:94,] %>%

mutate(PFA = case_when(AI == "Control" ~ "GlyControl", .default = PFA))

# first, determine significant differences in bee mortality

# syntax - cbind(successes, failures) as a function of [predictor] or [intercept]

b.clove <- glm(cbind(bees.dead, bees.alive) ~ PFA,

family = binomial, data = data_cage_clove)

b.clove.0 <- glm(cbind(bees.dead, bees.alive) ~ 1,

family = binomial, data = data_cage_clove)

b.fen <- glm(cbind(bees.dead, bees.alive) ~ PFA,

family = binomial, data = data_cage_fen)

b.fen.0 <- glm(cbind(bees.dead, bees.alive) ~ 1,

family = binomial, data = data_cage_fen)

b.oxalic <- glm(cbind(bees.dead, bees.alive) ~ PFA,

family = binomial, data = data_cage_oxalic)

b.oxalic.0 <- glm(cbind(bees.dead, bees.alive) ~ 1,

family = binomial, data = data_cage_oxalic)

# determine significance

anova(b.clove, b.clove.0)

# Resid. Df Resid. Dev Df Deviance Pr(>Chi)

# 1 25 94.932

# 2 30 107.499 -5 -12.567 0.0278 *

# Result: significant effect of treatment (P=0.0278)

anova(b.fen, b.fen.0)

# Resid. Df Resid. Dev Df Deviance Pr(>Chi)

# 1 25 306.46

# 2 30 454.11 -5 -147.66 < 2.2e-16 ***

# Result: significant effect of treatment (P<2.2e-16)

anova(b.oxalic, b.oxalic.0)

# Resid. Df Resid. Dev Df Deviance Pr(>Chi)

# 1 24 55.394

# 2 29 69.001 -5 -13.606 0.01831 *

# Result: significant effect of treatment (P=0.01831)

# glm summaries

summary(b.clove)

# Estimate Std. Error z value Pr(>|z|)

# (Intercept) -4.8798 0.2093 -23.314 <2e-16 ***

# PFAEco AE-13 -0.1960 0.3800 -0.516 0.6061

# PFAEco BC-12 0.5223 0.2765 1.889 0.0589 .

# PFAEco SE-11 0.1574 0.2901 0.543 0.5874

# PFAGlyControl -0.3260 0.3157 -1.033 0.3018

# PFAS-7500 0.5041 0.3073 1.640 0.1009

summary(b.fen)

# Estimate Std. Error z value Pr(>|z|)

# (Intercept) -5.0310 0.2896 -17.372 < 2e-16 ***

# PFAEco BC-12 1.5335 0.3158 4.856 1.20e-06 ***

# PFAEco CE-13 1.8560 0.3110 5.968 2.41e-09 ***

# PFAEco SE-11 1.9007 0.3124 6.084 1.17e-09 ***

# PFAGlyControl -1.4036 0.6463 -2.172 0.02988 *

# PFAS-7500 0.9765 0.3449 2.831 0.00463 **

summary(b.oxalic)

# Estimate Std. Error z value Pr(>|z|)

# (Intercept) -7.471 1.000 -7.471 7.93e-14 ***

# PFAEco BC-12 2.396 1.049 2.284 0.0224 *

# PFAEco CE-13 2.263 1.054 2.146 0.0319 *

# PFAEco SE-11 2.538 1.041 2.438 0.0148 *

# PFAGlyControl 1.841 1.080 1.704 0.0883 .

# PFAS-7500 2.183 1.054 2.071 0.0384 *

# Use emmeans to

# a) make point estimates for "Varroa mortality probability" for each treatment

# Std Err also provided

emmeans(b.clove, ~ PFA, type = "response")

# PFA prob SE df asymp.LCL asymp.UCL

# Control 0.00754 0.00157 Inf 0.00502 0.01132

# Eco AE-13 0.00621 0.00196 Inf 0.00334 0.01150

# Eco BC-12 0.01265 0.00226 Inf 0.00891 0.01793

# Eco SE-11 0.00882 0.00176 Inf 0.00596 0.01301

# GlyControl 0.00545 0.00128 Inf 0.00344 0.00864

# S-7500 0.01242 0.00276 Inf 0.00803 0.01918

emmeans(b.fen, ~ PFA, type = "response")

# PFA prob SE df asymp.LCL asymp.UCL

# Control 0.00649 0.001870 Inf 0.003689 0.01139

# Eco BC-12 0.02939 0.003590 Inf 0.023108 0.03730

# Eco CE-13 0.04012 0.004370 Inf 0.032382 0.04961

# Eco SE-11 0.04187 0.004700 Inf 0.033569 0.05212

# GlyControl 0.00160 0.000924 Inf 0.000517 0.00496

# S-7500 0.01705 0.003140 Inf 0.011873 0.02443

emmeans(b.oxalic, ~ PFA, type = "response")

# PFA prob SE df asymp.LCL asymp.UCL

# Control 0.000569 0.000568 Inf 8.02e-05 0.00402

# Eco BC-12 0.006211 0.001960 Inf 3.35e-03 0.01150

# Eco CE-13 0.005438 0.001810 Inf 2.83e-03 0.01042

# Eco SE-11 0.007151 0.002060 Inf 4.07e-03 0.01255

# GlyControl 0.003576 0.001460 Inf 1.61e-03 0.00794

# S-7500 0.005025 0.001670 Inf 2.62e-03 0.00963

# b) Pairwise comparisons between treatments, Tukey-corrected

b.tukey.clove <- emmeans(b.clove, pairwise ~ PFA, type = "response")$contrasts

data.frame(tukey.clove) %>%

arrange(p.value)

# contrast odds.ratio SE df null z.ratio p.value

# P < 0.05

# 1 (Eco BC-12) / GlyControl 2.3356749 0.6949618 Inf 1 2.85102739 0.04981395

# P > 0.05

# 2 GlyControl / (S-7500) 0.4360146 0.1422832 Inf 1 -2.54370655 0.11163906

# 3 (Eco AE-13) / (Eco BC-12) 0.4875985 0.1780206 Inf 1 -1.96732249 0.36125299

# 4 Control / (Eco BC-12) 0.5931562 0.1640372 Inf 1 -1.88862072 0.40910944

# 5 (Eco AE-13) / (S-7500) 0.4965646 0.1931205 Inf 1 -1.79999477 0.46567601

# 6 Control / (S-7500) 0.6040634 0.1856329 Inf 1 -1.64030181 0.57163332

# 7 (Eco SE-11) / GlyControl 1.6216056 0.5030010 Inf 1 1.55846877 0.62608708

# 8 (Eco BC-12) / (Eco SE-11) 1.4403471 0.3892319 Inf 1 1.35024840 0.75671458

# 9 (Eco SE-11) / (S-7500) 0.7070438 0.2132703 Inf 1 -1.14927263 0.86061607

# 10 Control / GlyControl 1.3854201 0.4373823 Inf 1 1.03262449 0.90707200

# 11 (Eco AE-13) / (Eco SE-11) 0.7023111 0.2636991 Inf 1 -0.94115561 0.93582632

# 12 Control / (Eco SE-11) 0.8543508 0.2478565 Inf 1 -0.54259705 0.99439882

# 13 Control / (Eco AE-13) 1.2164850 0.4623173 Inf 1 0.51563961 0.99559066

# 14 (Eco AE-13) / GlyControl 1.1388715 0.4505165 Inf 1 0.32872590 0.99949371

# 15 (Eco BC-12) / (S-7500) 1.0183884 0.2939247 Inf 1 0.06313342 0.99999986

b.tukey.fen <- emmeans(b.fen, pairwise ~ PFA, type = "response")$contrasts

data.frame(tukey.fen) %>%

arrange(p.value)

# contrast odds.ratio SE df null z.ratio p.value

# P < 0.05

# 1 Control / (Eco SE-11) 0.14947139 0.04669603 Inf 1 -6.0838754 1.757386e-08

# 2 Control / (Eco CE-13) 0.15629348 0.04860912 Inf 1 -5.9676826 3.603676e-08

# 3 (Eco SE-11) / GlyControl 27.22714211 16.05245716 Inf 1 5.6043952 3.123686e-07

# 4 (Eco CE-13) / GlyControl 26.03869969 15.33255013 Inf 1 5.5356301 4.632847e-07

# 5 (Eco BC-12) / GlyControl 18.86120168 11.15393658 Inf 1 4.9666203 1.009881e-05

# 6 Control / (Eco BC-12) 0.21576986 0.06813581 Inf 1 -4.8563643 1.767679e-05

# 7 (Eco SE-11) / (S-7500) 2.51972002 0.55669650 Inf 1 4.1828783 4.131401e-04

# 8 (Eco CE-13) / (S-7500) 2.40973631 0.52762967 Inf 1 4.0168416 8.358816e-04

# 9 GlyControl / (S-7500) 0.09254442 0.05621249 Inf 1 -3.9183795 1.252054e-03

# P > 0.05

# 10 Control / (S-7500) 0.37662606 0.12989361 Inf 1 -2.8313654 5.264211e-02

# 11 (Eco BC-12) / (S-7500) 1.74549893 0.39392256 Inf 1 2.4682859 1.335669e-01

# 12 Control / GlyControl 4.06967886 2.63032901 Inf 1 2.1716124 2.510765e-01

# 13 (Eco BC-12) / (Eco SE-11) 0.69273527 0.11914706 Inf 1 -2.1344061 2.694496e-01

# 14 (Eco BC-12) / (Eco CE-13) 0.72435267 0.12273881 Inf 1 -1.9031226 4.001034e-01

# 15 (Eco CE-13) / (Eco SE-11) 0.95635082 0.15595742 Inf 1 -0.2736797 9.997938e-01

b.tukey.oxalic <- emmeans(b.oxalic, pairwise ~ PFA, type = "response")$contrasts

data.frame(tukey.oxalic) %>%

arrange(p.value)

# c contrast odds.ratio SE df null z.ratio p.value

# P > 0.05

# 1 Control / (Eco SE-11) 0.07901728 0.08226590 Inf 1 -2.4378614 0.1432811

# 2 Control / (Eco BC-12) 0.09106433 0.09553512 Inf 1 -2.2840538 0.2004709

# 3 Control / (Eco CE-13) 0.10409159 0.10975129 Inf 1 -2.1458114 0.2637330

# 4 Control / (S-7500) 0.11269211 0.11881683 Inf 1 -2.0705626 0.3027951

# 5 Control / GlyControl 0.15860371 0.17135419 Inf 1 -1.7043318 0.5288327

# 6 (Eco SE-11) / GlyControl 2.00720288 1.00600383 Inf 1 1.3901566 0.7330724

# 7 (Eco BC-12) / GlyControl 1.74166667 0.90144081 Inf 1 1.0720068 0.8926116

# 8 (Eco SE-11) / (S-7500) 1.42617047 0.63075342 Inf 1 0.8026597 0.9671233

# 9 (Eco CE-13) / GlyControl 1.52369380 0.80479797 Inf 1 0.7973239 0.9680572

# 10 GlyControl / (S-7500) 0.71052632 0.37525918 Inf 1 -0.6470778 0.9873693

# 11 (Eco CE-13) / (Eco SE-11) 0.75911300 0.33577536 Inf 1 -0.6230804 0.9893705

# 12 (Eco BC-12) / (S-7500) 1.23750000 0.57017411 Inf 1 0.4624953 0.9973666

# 13 (Eco BC-12) / (Eco SE-11) 0.86770834 0.37276263 Inf 1 -0.3303107 0.9994817

# 14 (Eco BC-12) / (Eco CE-13) 1.14305556 0.52671971 Inf 1 0.2901586 0.9997251

# 15 (Eco CE-13) / (S-7500) 1.08262454 0.51169153 Inf 1 0.1679677 0.9999816

# no significant effects in oxalic acid

# determine significant differences in mite mortality

# syntax - cbind(successes, failures) as a function of [predictor] or [intercept]

m.clove <- glm(cbind(varroa.dead, varroa.wash) ~ PFA,

family = binomial, data = data_cage_clove)

m.clove.0 <- glm(cbind(varroa.dead, varroa.wash) ~ 1,

family = binomial, data = data_cage_clove)

m.fen <- glm(cbind(varroa.dead, varroa.wash) ~ PFA,

family = binomial, data = data_cage_fen)

m.fen.0 <- glm(cbind(varroa.dead, varroa.wash) ~ 1,

family = binomial, data = data_cage_fen)

m.oxalic <- glm(cbind(varroa.dead, varroa.wash) ~ PFA,

family = binomial, data = data_cage_oxalic)

m.oxalic.0 <- glm(cbind(varroa.dead, varroa.wash) ~ 1,

family = binomial, data = data_cage_oxalic)

# determine significance

anova(m.clove, m.clove.0)

# Resid. Df Resid. Dev Df Deviance Pr(>Chi)

# 1 25 50.928

# 2 30 128.030 -5 -77.102 3.386e-15 ***

# Result: significant effect of treatment (P=3.386e-15)

anova(m.fen, m.fen.0)

# Resid. Df Resid. Dev Df Deviance Pr(>Chi)

# 1 25 79.083

# 2 30 212.911 -5 -133.83 < 2.2e-16 ***

# Result: significant effect of treatment (P<2.2e-16)

anova(m.oxalic, m.oxalic.0)

# Resid. Df Resid. Dev Df Deviance Pr(>Chi)

# 1 24 42.746

# 2 29 141.448 -5 -98.702 < 2.2e-16 ***

# Result: significant effect of treatment (P<2.2e-16)

# glm summaries

summary(m.clove)

# Estimate Std. Error z value Pr(>|z|)

# (Intercept) -0.6286 0.1787 -3.517 0.000436 ***

# PFAEco AE-13 1.6362 0.3022 5.415 6.12e-08 ***

# PFAEco BC-12 1.1646 0.2465 4.724 2.31e-06 ***

# PFAEco SE-11 0.4704 0.2417 1.946 0.051649 .

# PFAGlyControl -0.3087 0.2481 -1.244 0.213355

# PFAS-7500 0.9963 0.3076 3.239 0.001200 **

summary(m.fen)

# Estimate Std. Error z value Pr(>|z|)

# (Intercept) 0.1292 0.1800 0.718 0.47281

# PFAEco BC-12 1.5210 0.2865 5.310 1.10e-07 ***

# PFAEco CE-13 1.5369 0.2862 5.369 7.90e-08 ***

# PFAEco SE-11 1.9139 0.3363 5.691 1.26e-08 ***

# PFAGlyControl -0.8505 0.2736 -3.109 0.00188 **

# PFAS-7500 0.8390 0.2800 2.997 0.00273 **

summary(m.oxalic)

# Estimate Std. Error z value Pr(>|z|)

# (Intercept) 0.8650 0.2433 3.555 0.000378 ***

# PFAEco BC-12 1.8918 0.5702 3.318 0.000906 ***

# PFAEco CE-13 0.5473 0.3531 1.550 0.121136

# PFAEco SE-11 1.1066 0.4035 2.743 0.006094 **

# PFAGlyControl -1.8011 0.3589 -5.018 5.22e-07 ***

# PFAS-7500 0.5213 0.3370 1.547 0.121875

# Use emmeans to

# a) make point estimates for "Varroa mortality probability" for each treatment

# Std Err also provided

emmeans(m.clove, ~ PFA, type = "response")

# PFA prob SE df asymp.LCL asymp.UCL

# Control 0.348 0.0405 Inf 0.273 0.431

# Eco AE-13 0.733 0.0477 Inf 0.630 0.815

# Eco BC-12 0.631 0.0395 Inf 0.551 0.704

# Eco SE-11 0.461 0.0404 Inf 0.383 0.540

# GlyControl 0.281 0.0348 Inf 0.218 0.354

# S-7500 0.591 0.0605 Inf 0.469 0.702

emmeans(m.fen, ~ PFA, type = "response")

# PFA prob SE df asymp.LCL asymp.UCL

# Control 0.532 0.0448 Inf 0.444 0.618

# Eco BC-12 0.839 0.0301 Inf 0.771 0.890

# Eco CE-13 0.841 0.0298 Inf 0.774 0.891

# Eco SE-11 0.885 0.0289 Inf 0.816 0.931

# GlyControl 0.327 0.0454 Inf 0.245 0.421

# S-7500 0.725 0.0428 Inf 0.634 0.800

emmeans(m.oxalic, ~ PFA, type = "response")

# PFA prob SE df asymp.LCL asymp.UCL

# Control 0.704 0.0507 Inf 0.596 0.793

# Eco BC-12 0.940 0.0289 Inf 0.851 0.977

# Eco CE-13 0.804 0.0403 Inf 0.713 0.871

# Eco SE-11 0.878 0.0345 Inf 0.793 0.931

# GlyControl 0.282 0.0534 Inf 0.190 0.397

# S-7500 0.800 0.0373 Inf 0.717 0.863

# b) Pairwise comparisons between treatments, Tukey-corrected

tukey.clove <- emmeans(m.clove, pairwise ~ PFA, type = "response")$contrasts

data.frame(tukey.clove) %>%

arrange(p.value)

# contrast odds.ratio SE df null z.ratio p.value

# P < 0.05

# 1 (Eco AE-13) / GlyControl 6.9935245 2.08591414 Inf 1 6.5210248 1.046951e-09

# 2 (Eco BC-12) / GlyControl 4.3636364 1.05479159 Inf 1 6.0950149 1.639323e-08

# 3 Control / (Eco AE-13) 0.1947090 0.05883168 Inf 1 -5.4153213 9.129818e-07

# 4 Control / (Eco BC-12) 0.3120567 0.07692345 Inf 1 -4.7243331 3.400667e-05

# 5 GlyControl / (S-7500) 0.2711538 0.08237366 Inf 1 -4.2959662 2.514449e-04

# 6 (Eco AE-13) / (Eco SE-11) 3.2086957 0.94005535 Inf 1 3.9794512 9.756909e-04

# 7 (Eco SE-11) / GlyControl 2.1795537 0.51619436 Inf 1 3.2897186 1.285400e-02

# 8 Control / (S-7500) 0.3692308 0.11357823 Inf 1 -3.2389741 1.521212e-02

# 9 (Eco BC-12) / (Eco SE-11) 2.0020779 0.47081174 Inf 1 2.9519520 3.723658e-02

# P > 0.05

# 10 Control / (Eco SE-11) 0.6247619 0.15101321 Inf 1 -1.9460444 3.739353e-01

# 11 (Eco AE-13) / (S-7500) 1.8963211 0.66243611 Inf 1 1.8318532 4.450689e-01

# 12 (Eco SE-11) / (S-7500) 0.5909944 0.17646789 Inf 1 -1.7614126 4.909583e-01

# 13 (Eco AE-13) / (Eco BC-12) 1.6026827 0.47589528 Inf 1 1.5884831 6.062031e-01

# 14 Control / GlyControl 1.3617021 0.33784039 Inf 1 1.2443917 8.149052e-01

# 15 (Eco BC-12) / (S-7500) 1.1832168 0.35790729 Inf 1 0.5561793 9.937130e-01

tukey.fen <- emmeans(m.fen, pairwise ~ PFA, type = "response")$contrasts

data.frame(tukey.fen) %>%

arrange(p.value)

# contrast odds.ratio SE df null z.ratio p.value

# P < 0.05

# 1 (Eco BC-12) / GlyControl 10.7142857 3.25204578 Inf 1 7.81347054 1.175726e-13

# 2 (Eco SE-11) / GlyControl 15.8693878 5.56896020 Inf 1 7.87745042 1.367795e-13

# 3 (Eco CE-13) / GlyControl 10.8857143 3.30181808 Inf 1 7.87115228 1.406653e-13

# 4 Control / (Eco SE-11) 0.1475096 0.04960375 Inf 1 -5.69136404 1.884827e-07

# 5 GlyControl / (S-7500) 0.1845992 0.05490138 Inf 1 -5.68096718 2.002949e-07

# 6 Control / (Eco CE-13) 0.2150421 0.06155373 Inf 1 -5.36933879 1.178754e-06

# 7 Control / (Eco BC-12) 0.2184828 0.06258666 Inf 1 -5.30980258 1.635839e-06

# 8 Control / GlyControl 2.3408867 0.64045057 Inf 1 3.10873934 2.311571e-02

# 9 (Eco SE-11) / (S-7500) 2.9294756 1.04266037 Inf 1 3.01984143 3.040177e-02

# 10 Control / (S-7500) 0.4321257 0.12098320 Inf 1 -2.99686403 3.258216e-02

# P > 0.05

# 11 (Eco CE-13) / (S-7500) 2.0094937 0.62110006 Inf 1 2.25791485 2.115685e-01

# 12 (Eco BC-12) / (S-7500) 1.9778481 0.61172200 Inf 1 2.20510472 2.352239e-01

# 13 (Eco BC-12) / (Eco SE-11) 0.6751543 0.24376203 Inf 1 -1.08798758 8.863857e-01

# 14 (Eco CE-13) / (Eco SE-11) 0.6859568 0.24754249 Inf 1 -1.04452772 9.028340e-01

# 15 (Eco BC-12) / (Eco CE-13) 0.9842520 0.31001175 Inf 1 -0.05039607 1.000000e+00

tukey.oxalic <- emmeans(m.oxalic, pairwise ~ PFA, type = "response")$contrasts

data.frame(tukey.oxalic) %>%

arrange(p.value)

# c ontrast odds.ratio SE df null z.ratio p.value

# P < 0.05

# 1 (Eco SE-11) / GlyControl 18.31363636 7.62109274 Inf 1 6.98713061 4.212097e-11

# 2 GlyControl / (S-7500) 0.09803922 0.03451697 Inf 1 -6.59632203 6.320026e-10

# 3 (Eco CE-13) / GlyControl 10.46842105 3.84717671 Inf 1 6.39005085 2.485842e-09

# 4 (Eco BC-12) / GlyControl 40.16250000 23.26227049 Inf 1 6.37588024 2.726890e-09

# 5 Control / GlyControl 6.05625000 2.17366493 Inf 1 5.01818656 7.740016e-06

# 6 Control / (Eco BC-12) 0.15079365 0.08597614 Inf 1 -3.31810566 1.168283e-02

# P < 0.05

# 7 Control / (Eco SE-11) 0.33069620 0.13342175 Inf 1 -2.74268314 6.712450e-02

# 8 (Eco BC-12) / (S-7500) 3.93750000 2.22814070 Inf 1 2.42198569 1.485545e-01

# 9 (Eco BC-12) / (Eco CE-13) 3.83653846 2.20832630 Inf 1 2.33593038 1.796428e-01

# 10 Control / (Eco CE-13) 0.57852564 0.20426226 Inf 1 -1.55002262 6.316548e-01

# 11 Control / (S-7500) 0.59375000 0.20008416 Inf 1 -1.54694928 6.336772e-01

# 12 (Eco SE-11) / (S-7500) 1.79545455 0.71348673 Inf 1 1.47277375 6.817706e-01

# 13 (Eco CE-13) / (Eco SE-11) 0.57161892 0.23500272 Inf 1 -1.36039527 7.507832e-01

# 14 (Eco BC-12) / (Eco SE-11) 2.19303797 1.33295645 Inf 1 1.29198965 7.896171e-01

# 15 (Eco CE-13) / (S-7500) 1.02631579 0.35522730 Inf 1 0.07504787 9.999997e-01

# Field Trials------------------------------

# Load the data

data1 <- S5_Dataset

data1 <- read_excel('S5_Dataset.xlsx', sheet = "Field Summary")

# Calculate the difference between WashFinal and WashInitial

data1 <- data1 %>%

rename(

wash_initial = WashInitial,

wash_final = WashFinal

) %>%mutate(washdiff = wash_final - wash_initial)

hist(data1$washdiff)

shapiro.test(data1$washdiff)

data1_long<- data1%>%

pivot_longer(

cols = c(wash_initial, wash_final),

names_to = c(".value", "day"),

names_sep = "_"

) %>%

mutate(

wash_r = round(wash),

day = factor(day, levels = c("initial", "final"))

)

# '''

# To assess treatment efficacy over time, we calculated the difference in

# mite counts between initial and final washes (washdiff) for each colony.

#

# A Shapiro-Wilk test indicated that washdiff was not normally distributed ,

# and the histogram revealed skewed residuals.

#

# To accommodate the count-based and overdispersed nature of the data,

# we modeled individual wash counts using a generalized linear mixed model (GLMM)

# with a negative binomial distribution and a random intercept for colony.

# '''

m1_wash <- glmer.nb(wash_r ~ Treatment + day + Treatment:day + (1|Colony) , data = data1_long)

summary(m1_wash)

#This model included fixed effects for Treatment, day (initial vs. final), and their interaction, with random intercepts for Colony

m1_wash_pos <- glmer(wash_r ~ Treatment + day + Treatment:day + (1|Colony),family = poisson, data = data1_long)

anova(m1_wash,m1_wash_pos)

#To confirm the presence of overdispersion and justify using a negative binomial distribution,

# we compared the above model to a Poisson GLMM.

#The negative binomial model fit significantly better

# than the Poisson model (very small p-value),

# indicating substantial overdispersion and supporting the use of the negative binomial distribution.

m1_wash.0 <- glmer.nb(wash_r ~ Treatment + day + (1|Colony) , data = data1_long)

anova(m1_wash,m1_wash.0)

#We tested whether the interaction term improved the model fit

# by comparing the full model with one omitting the interaction.

# This comparison showed no significant improvement from including the interaction,

# suggesting that while the fixed effects for Treatment and day are important,

# their interaction may not be necessary in the final model.

residuals <- resid(m1_wash, type = "pearson")

fitted_vals <- fitted(m1_wash)

plot(fitted_vals, residuals,

xlab = "Fitted values",

ylab = "Pearson residuals",

main = "Residuals vs Fitted")

abline(h = 0, col = "red")

shapiro.test(residuals)

#Residual vs. Fitted plot showed no obvious pattern or heteroscedasticity.

#Shapiro-Wilk test of Pearson residuals showed no significant deviation from normality, indicating adequate model fit despite the count nature of the data.

emm <- emmeans(m1_wash, ~ day | Treatment)

contrast(emm, method = "revpairwise")

'''

Oxalic-treated colonies had an estimated

exp(-0.569) ≈ 0.566 times as many mites after treatment compared to before —

indicating a 43.4% reduction in Varroa load.

However, this reduction was not statistically significant (z = –1.576, p = 0.115).

Adjuvant-treated colonies showed an estimated

exp(-0.433) ≈ 0.648 times as many mites post-treatment —

a 35.2% reduction, also not statistically significant (z = -1.155, p = 0.248).

Control colonies had exp(0.471) ≈ 1.602 times as many mites post-treatment,

suggesting a 60% increase in Varroa load, but this increase was likewise not significant (z = 1.203, p = 0.229).

'''

##

data2 <- read_excel('S6_Dataset.xlsx')

data2 <- data2 %>%

rename(wash_final = PostMitesPer100,

wash_initial = PreMitesPer100,

)%>%mutate(wash_diff =wash_final-wash_initial)

data2_long<-data2%>%

pivot_longer(c(wash_final, wash_initial), names_to = c(".value", "day"), names_sep = "_") %>%

mutate(wash_r = round(100*wash), # Multiply by 100 and round this because technically negative binomial is supposed to be for count data

day = factor(day, levels = c("initial", "final"))

)

hist(data2$wash_diff)

shapiro.test(data2$wash_diff)

m2_wash_diff <- lmer(wash_diff ~ Treatment + day + Treatment:day + (1|Colony) , data = data2_long)

residuals <- resid(m2_wash_diff, type = "pearson")

fitted_vals <- fitted(m2_wash_diff)

plot(fitted_vals, residuals,

xlab = "Fitted values",

ylab = "Pearson residuals",

main = "Residuals vs Fitted")

abline(h = 0, col = "red")

cor.test(fitted_vals, residuals)

#To evaluate changes in mite infestation after treatment,

# we calculated the difference in mite counts per 100 bees for each colony.

# A Shapiro-Wilk test indicated no significant deviation from normality.

# However, when we fit a linear mixed-effects model using wash_diff as the response,

# diagnostic plots and a correlation test revealed

# a significant correlation between the residuals and fitted values —

# suggesting non-constant variance and potential model misfit.

# Therefore, despite approximate normality,

# we adopted a negative binomial generalized linear mixed model (GLMM)

# to appropriately account for the count-based nature of the data and overdispersion.

m2_wash <- glmer.nb(wash_r ~ Treatment + day + Treatment:day + (1|Colony) , data = data2_long)

summary(m2_wash)

m2_wash.0 <- glmer.nb(wash_r ~ Treatment + day + (1|Colony) , data = data2_long)

anova(m2_wash,m2_wash.0)

'''

To determine whether the effect of treatment on mite counts differed over time,

we compared two nested negative binomial generalized linear mixed models (GLMMs):

A main-effects model that included Treatment and day as additive fixed effects.

A full model that included an additional Treatment × day interaction term.

The full model provided a substantially better fit,

with a much lower AIC and

a highly significant likelihood ratio test (χ²(2) = 287, p < 2.2 × 10⁻¹⁶).

This result strongly supports the presence of

a significant interaction between treatment and time point,

indicating that the effect of treatment on mite counts differs before and after treatment.

'''

residuals <- resid(m2_wash, type = "pearson")

fitted_vals <- fitted(m2_wash)

plot(fitted_vals, residuals,

xlab = "Fitted values",

ylab = "Pearson residuals",

main = "Residuals vs Fitted")

abline(h = 0, col = "red")

shapiro.test(residuals)

'''

A Shapiro-Wilk test indicated no evidence

of non-normality in the model residuals (W = 0.987, p = 0.827),

suggesting that the residuals were approximately normally distributed

and that the model fit the data adequately.

The residuals vs. fitted values plot showed

no clear patterns, curvature, or heteroscedasticity,

suggesting that the model adequately captured the structure of the data

and that residual variance was relatively constant across predicted values.

'''

emm <- emmeans(m2_wash, ~ day | Treatment)

contrast(emm, method = "revpairwise")

'''

We compared the estimated mite counts per 100 bees before and after treatment

for each treatment group, using a negative binomial mixed-effects model:

In the Adjuvant group, colonies had

approximately 29% fewer mites after treatment compared to before.

This reduction was statistically significant (p < 0.0001),

suggesting that the treatment was effective at lowering mite infestation.

In the Control group, colonies had

about 2.6 times more mites after treatment than before — a 162% increase.

This increase was also statistically significant (p < 0.0001),

indicating that mite loads worsened without intervention.

In the Oxalic acid only group, mite counts increased

by about 184% after treatment, or roughly 2.8 times as many mites post-treatment compared to baseline.

This increase was statistically significant (p < 0.0001),

suggesting that oxalic acid alone was not effective in reducing mite loads.

'''

'''

In Field Trial 1, neither oxalic acid alone nor oxalic acid

combined with an adjuvant resulted in

a statistically significant reduction in mite counts.

The control group also showed no significant increase in mite load over time.

However, the lack of significance across all groups may be attributed

to high variability and limited sample size,

potentially reducing the statistical power to detect treatment effects.

In Field Trial 2, the results were more definitive:

Colonies treated with oxalic acid alone experienced a significant 184% increase in mite counts

following treatment, indicating that oxalic acid without an adjuvant was ineffective

under these field conditions.

In contrast, colonies treated with oxalic acid combined with a bee-safe adjuvant

showed a significant 29% reduction in mite loads, demonstrating clear treatment efficacy

when the adjuvant was included.

'''

#mite drop

data1 <- data1 %>%

mutate(

baseline = `-2`,

drop_0= round(`0`),

drop_0to2 = round(`0` + `2`),

drop_0to4 = round(`0` + `2` + `4`),

drop_0to7 = round(`0` + `2` + `4` + `7`),

drop_0to14 = round(`0` + `2` + `4` + `7` + `14`),

drop_0to21 = round(`0` + `2` + `4` + `7` + `14` + `21`)

)

print(data1,width=Inf)

# Start with base model

base_model <- glm.nb(drop_0to21 ~ Treatment, data = data1)

# Full model with all potential predictors

full_model <- glm.nb(drop_0to21 ~ Treatment + baseline + Location + SeamsInitial + wash_initial, data = data1)

# Perform forward selection

step_model <- stepAIC(base_model,

scope = list(lower = base_model, upper = full_model),

direction = "forward")

summary(step_model)

res <- resid(step_model)

shapiro.test(res)

plot(fitted(step_model), res,

xlab = "Fitted values",

ylab = "Residuals",

main = "Residuals vs Fitted")

abline(h = 0, col = "red")

cor.test(fitted(step_model), res)

'''

To analyze cumulative mite drop over the full 23-day treatment period,

we modeled the data using a negative binomial generalized linear model

to account for overdispersion in count data. We began with a base model

including only Treatment, and used forward selection to evaluate the

added value of additional predictors: Location, SeamsInitial,wash_initial

and baseline.

The final model includes all predictors. And diagnostics support the

adequacy of the model for inference.

Adjuvant vs. Control:

Colonies treated with the Adjuvant had significantly greater mite drop

compared to the Control group. Specifically, mite drop was approximately

_exp(1.55) ≈ 4.7 times higher in the Adjuvant group than in the Control group.

This 370% increase in cumulative mite drop over 23 days was statistically

significant (p < 0.001), highlighting the strong efficacy of the Adjuvant

treatment relative to no treatment.

Adjuvant vs. Oxalic:

Compared to colonies treated with Oxalic acid alone, the Adjuvant group

experienced an approximately _exp(0.10) ≈ 1.11 times higher mite drop — an

11% increase. However, this difference was not statistically significant

(p = 0.581), suggesting that while the direction of the effect favors Adjuvant,

the improvement over Oxalic alone during the full treatment period was not

strong enough to reach significance.

'''

'''

We applied the same forward selection approach using AIC to identify

the best-fitting negative binomial generalized linear model for analyzing

cumulative mite drop counts over the first 2 and 4 days of treatment.

'''

base_model_2 <- glm.nb(drop_0to2 ~ Treatment + baseline, data = data1)

# Full model

full_model_2 <- glm.nb(drop_0to2 ~ Treatment + baseline + Location + SeamsInitial + wash_initial, data = data1)

# Forward selection

step_model_2 <- stepAIC(base_model_2,

scope = list(lower = base_model_2, upper = full_model_2),

direction = "forward")

# Summary

summary(step_model_2)

'''

In the first 4 days, colonies treated with Adjuvant had

about 6.42 times more mite drop than those in the Control group (exp(1.86)),

a highly significant difference (p < 0.001).

Compared to Oxalic, Adjuvant-treated colonies had about 1.46 times more mite drop,

but this difference was not statistically significant (p = 0.117).

'''

##

base_model_0<- glm.nb(drop_0 ~ Treatment + baseline, data = data1)

# Full model

full_model_0 <- glm.nb(drop_0 ~ Treatment + baseline + Location + SeamsInitial + wash_initial, data = data1)

# Forward selection

step_model_0 <- stepAIC(base_model_0,

scope = list(lower = base_model_0, upper = full_model_0),

direction = "forward")

# Summary

summary(step_model_0)

'''

In first 2 days, colonies treated with Adjuvant had

about 8.51 times more mite drop than those in the Control group (exp(2.14),

p < 0.001),

and approximately 2.75 times more than those treated with Oxalic acid alone

(exp(1.01), p < 0.001), indicating that the Adjuvant formulation had a

significantly stronger immediate impact on mite removal.

'''
