# Supplementary material for: Adjuvants to improve efficacy of miticides in managed honey bee (Apis mellifera) colonies to control Varroa destructor
Source: PLoS One. 2025 Jun 17;20(6):e0320037. doi: 10.1371/journal.pone.0320037 (PMC12173398; doi:10.1371/journal.pone.0320037)
Supplement: Attachment — Submitted filename: Comments to Author_FINAL.docx [file pone.0320037.s007.docx]

## Overall

This seems to be a well-conducted study. Appropriate methods are used for the LC50 analysis. A few more key details are needed: sample size for cage trials (number of cages per treatment), and initial and ending mite levels for the field experiment.

I have some suggestions (in blue text) for alternative statistical approaches for some the analyses, mostly to incorporate the “generalized” version of the linear modeling. I think reanalyses will let you say more about the effect of adjuvants in cage trials, but likely you will need to pull back some of your statements about significant effects in the field trial.

I appreciate you including your data, as this allowed me to test some alternative approaches. At the end of this document I have included the R code I used when reviewing your analyses. If you paste this into an R script, some of this code may be helpful, or at least will let you follow my process of reviewing your stats.

## Abstract

- Nice and clear

## Introduction

- L38-39. Remove the word “average” to reflect the kind of estimate that’s made, an estimate of the loss rate for the population of honey bee colonies. For the CAPA survey please confirm, but you will probably remove “average” here as well.
- L41-42. This is a little different than what you have written. 60% of commercial beekeeping operations reported it as a leading cause of winter loss.
- L42-43. This list of pathogens may not be necessary since “associated diseases” was already mentioned.
- L67. Spelling of Mitchondrial
- L84. Reconsider word choice of “greaten”
- L93. Capitalization and italicization of “Varroa”

## Materials and Methods

- Data analysis methods for the LC50 determination should be briefly added. Reading the manuscript I think you used the drc package to fit 2-parameter log-logistic dose-response models.
- L97. Do you have an analysis of the chemical constituents of the clove oil used? Or if not, any reference for usual composition and / or how much these vary?
- L99. How is 102.5% purity possible? Spelling of Scientific
- L127 and L134-136. Only one of these statements is needed.
- L133. “Sonicating” or rephrase
- L152. Were the weigh boats or trays under the cages covered with Vaseline as in the Rinkevich protocol? I am only asking since it is not mentioned here or on L180
- L170. Replace “phoretic mites” with “mites in the dispersal phase” or “mites on adult bees” as phoresy refers to non-feeding transport.
- L176. The description of cage treatment series could be more specific. “Four active ingredient-adjuvant combinations” is ambiguous and could be a concentration range of one adjuvant, different adjuvants, or both. This could be your place to describe the blocking to limit confounding effects. Sample size is needed here.
- L187. Kruskal-Wallace test for differences in bee and Varroa mortality in cages
  - Why not run logistic regression instead of doing a nonparametric test?
  - I tried this on the clove data and found significant differences between treatments. Logistic regression seems to give you more power to detect differences.
  - Code provided at end of comments.
  - Please let me know if you see any issues with this approach
- L188. “Wilcoxon”
- L198-199. Great that you stratified. Great that you described the spatial arrangement of treatments. For completeness, specify whether the stratification was based on pre-treatment alcohol wash or sticky board mite fall.
- L213. “manila”
- L213. Closed or open? Perhaps specify dimensions as you do for the complete Swedish sponge.
- L235-236. I looked into the paired t test.
  - For year 1 the assumption of normally distributed deltas is violated, so the t test is not appropriate. I recommend generalized linear modeling (GLM) using a negative binomial distribution
  - The GLM I tried did not support a treatment x time interaction. This could be further finessed by accounting for both the bee number and the varroa number per sample. Code for my quick analysis, and a finessed analysis are provided at end of comments.
  - Unless a more finessed analysis gives different results, this calls for the removal of statements like on Line 24-25 “oxalic acid and adjuvant in glycerin caused a significant decrease in Varroa in Year 1”. This statement also highlights the OA-adjuvant effect over the OA effect even though OA had a better estimated effect, but more variation. This highlights statistical significance over biological significance, which can lead to a bottom-line conclusion that is not supported. Readers may think the adjuvant increased performance in year 1.
  - For year 2, the paired t test may be OK as the normality of deltas looks better, graphically. That said, the values fail a normality test, so the significant difference between control and OA-adjuvant should be re-evaluated as well.
  - On a different note, Figure S1B does a great job of showing patterns in the data, which are suggestive of a benefit of the adjuvant, even if not statistically significant.
- L239-240. Mixed modeling procedure
  - I am glad you accounted for the baseline mite drop. Is there a good theoretical rationale for including all the predictors? Maybe that is all you need as a covariate (see my code). Check for collinearity, for example by testing the model with `vif()` from the `car`package.
  - As our experience is that mite drop is usually not appropriately modeled with a normal distribution, consider a generalized procedure. I again recommend starting with a negative binomial distribution. That said, when modeled with a normal distribution, the residuals were not significantly non-normal, though by eye the histogram did not look great. You could make an argument that this is a case where the data allow the assumption of normality.

## Results

- Initial (and preferably ending) Varroa infestations are needed for context.
- L248. “was”
- L260. Move figure reference. Fig 1A refers to efficacy against Varroa, not effects on bee mortality. Check the other references in the statement.
- L266. Move figure reference. Fig 1A refers to efficacy against Varroa, not effects on bee mortality. Check the other references in the statement.
- L269-270. Move figure reference. Fig 1A refers to efficacy against Varroa, not effects on bee mortality. Check the other references in the statement.
- L283-290. By presenting only deltas, we lack important context. How high were pre-and post-treatment mite levels in the alcohol wash for both years?
- Figure 2. I think it would be more informative if you replace Figure 2 (showing the deltas) with Figure S1B. I think Figure S1B is excellent. You could remove Figure 2 or move it to the supplemental materials.
  - If you take my earlier suggestion to remove mentions of “significant decrease of Varroa per 100 bees in adjuvant group for year 1” this would necessitate removing the dagger in Pane A of Figure S1B
  - For the Figure S1B legend, it should say “a significant difference between treatments”
  - Additionally, you may need to remove significance between treatments
- Figure 3. Add baseline mite fall measurements.
- L301. The units (“Varroa per 100 bees”) are best placed with the number, i.e., following “1.3”
- L313. Results for analysis of mite fall. This usually needs to be a generalized model, not just a mixed model – initial suggestion: negative binomial distribution.

## Discussion / Conclusion

- The Discussion could be made more focused by reducing limitations, literature gaps, and future steps.
- I think the Discussion should be more focused on answering the “aim of the research” statement at the end of the Introduction. What did your research reveal about the usefulness of adjuvants for Varroa control? What uncertainties remain? Do you have any speculations about the impact of variables that changed between the two years of the field study? Any further explanation for why the oxalic-adjuvant combination was pursued and not the other active ingredients. Your suggestions for “next steps forward” are good, but they will be stronger if you can focus on how results from this study indicate which next steps should be pursued. With so many variables that can be manipulated, perhaps you have ideas for how to approach this systematically.
- Your Discussion may benefit from subheadings if the journal style allows.
- In the current form, I don’t think having a separate Conclusions section is helpful. The content from the Conclusion would be better at the start of the Discussion.
- L401-410. This content would be good to start your Discussion with.
- L330-343. This is a long discussion of limitations. It is clear that you had appropriate procedures in place to address them. Since you blocked all your cage trial treatments within cohorts of shaken bees, this section seems unnecessary here but could briefly be added to the Materials and Methods.
- L344-349. I think this can also be removed. The main thing is that you used the same method for all groups and timepoints that you compared. This point is made in the BEEBOOK Varroa standard methods paper (Dietemann et al. 2013, Section 4.2.3.1.2.4. Assessing the efficiency of dislodging method), so you could cite this in your Methods. There you may want to simply disclose that you did not fully evaluate the recovery efficacy of your alcohol wash but state that the same methods were used at all time points.
- L350-360. This is the first part that truly seems to be discussing your results. Keep this.
- L361-370. These are good Discussion points. They would be better if they were more connected to your results.
- L371-374. This belongs in the Introduction, or can be removed.
- L375-380. This could be part of a “next steps” paragraph
- 381-399. As written, this belongs in an Introduction.

Funding

- L426-427. There was a recent notice that USDA-NIFA funding should be acknowledged in a specific format: <https://content.govdelivery.com/accounts/USDANIFA/bulletins/3d43d38>

## Code used when reviewing analyses

library(MASS)

library(tidyverse)

library(lme4)

library(emmeans)

library(car)

\# Logistic regression to assess differences in Varroa mortality in cages ----------

\# As a demo, run this on the clove oil subset of data

\# data import

dat_cage <- read.csv("S4_Dataset.csv")

\# data prep

dat_cage_clove <- dat_cage %>%

filter(AI == "Clove Oil") # Omits GlyOnly controls, I need to approach data prep differently

\# second try to filter the data that are relevant to the clove subset of data

dat_cage_clove <- dat_cage[1:31,]

dat_cage_clove <- dat_cage_clove %>%

mutate(

PFA = case_when(AI == "Control" ~ "GlyControl", .default = PFA)

)

\# syntax - cbind(successes, failures) as a function of [predictor] or [intercept]

m.1 <- glm(cbind(X24h.varroa.dead, Varroa.Wash) ~ PFA, family = binomial, data = dat_cage_clove)

m.1.0 <- glm(cbind(X24h.varroa.dead, Varroa.Wash) ~ 1, family = binomial, data = dat_cage_clove)

anova(m.1, m.1.0)

\# Result: significant effect of treatment (P=3.386e-15)

summary(m.1)

\# Use emmeans to

\# a) make point estimates for "Varroa mortality probability" for each treatment

emmeans(m.1, ~ PFA, type = "response") # Confidence intervals also provided

\# b) Pairwise comparisons between treatments, Tukey-corrected

emmeans(m.1, pairwise ~ PFA, type = "response")$contrasts

\# here we are seeing significant differences between treatments

\# Pre and post Varroa infestations - potential finessed approach ---------

\# Some reorganizing of original data would be necessary to try this

```
## A model setup you could try -
## The response variable is the number of Varroa in the final sample (integer).
## There is an offset for the "denominator" of these samples,
## i.e., the number of bees in the final samples (may need to be integer).
## This is transformed by the link function (log),
## The covariate is the initial percent varroa infestation (can be decimal)
## Finessed model
```

\# m <- glm.nb(num_varroa_final ~ Treatment + percent_varroa_initial +

\#	offset(log(num_bees_final)), data=dat)

```
## To get emmeans predictions from finessed model you need to include what value
## of the offset you want to make predictions on. This would usually be
## mites per 100 bees, by setting offset = log(100)
```

\# preds <- emmip(m, ~ Treatment, type = "response", CIs = TRUE, plotit = FALSE, offset = log(100))

\# Pre and post Varroa infestations - Year 1 ---------

\# Demo on S5, Year 1 field trial dataset

\# Description: paired t test

dat_field1 <- read.csv("S5_Dataset.csv")

\# data prep

dat_field1_sub_control <- dat_field1 %>%

filter(Treatment == "Control")

dat_field1_sub_oxalic <- dat_field1 %>%

filter(Treatment == "Oxalic")

dat_field1_sub_adjuvant <- dat_field1 %>%

filter(Treatment == "Adjuvant")

\# I assume something like this was done

\# t.test(x, y, paired = TRUE, alternative = "two.sided")

t.test(dat_field1_sub_adjuvant$WashFinal, dat_field1_sub_adjuvant$WashInitial, paired = TRUE, alternative = "less")

\# This lines up with the reported values

\# Let's look at if differences between pairs are normally distributed

dat_field1 <- dat_field1 %>%

mutate(wash_delta = WashFinal - WashInitial)

hist(dat_field1$wash_delta) # The assumption of normality of differences between pairs does not hold.

\# What else can we do?

\# a) Use glmer.nb to run negative binomial mixed model, then pull out estimates and contrasts using emmeans

\# b) Or do a before-after-control-impact (BACI) analysis with negative binomial distribution (a contrast of contrasts analysis)

\# Data prep for mixed modeling

dat_field1_long <- dat_field1 %>%

rename(wash_final = WashFinal,

wash_initial = WashInitial

) %>%

pivot_longer(c(wash_final, wash_initial), names_to = c(".value", "day"), names_sep = "_") %>%

mutate(wash_r = round(wash), # Rounding this because technically negative binomial is supposed to be for count data

day = factor(day, levels = c("initial", "final"))

)

\# Mixed modeling, separately for each treatment

\# Just looking at effect of day within treatment, as with the initial paired t tests

m.2 <- glmer.nb(wash_r ~ day + (1|Colony), data = dat_field1_long, subset = (Treatment == "Adjuvant"))

summary(m.2) # almost significant reduction, P = 0.056

\# What is the estimated percent reduction?

exp(-0.3545) # Results: The final Varroa level was 0.7 times as high after treatment as before treatment. A 30% reduction.

m.3 <- glmer.nb(wash_r ~ day + (1|Colony), data = dat_field1_long, subset = (Treatment == "Control"))

summary(m.3) # Not significant

exp(0.4639) # Results: Final Varroa level was 1.59 times as high after treatment

m.4 <- glmer.nb(wash_r ~ day + (1|Colony), data = dat_field1_long, subset = (Treatment == "Oxalic"))

summary(m.4) # Not significant

exp(-0.4339) # Results: Oxalic treated colonies had 0.65 times as many Varroa after treatment as before

\# Mixed modeling, all treatments modeled together

m.5 <- glmer.nb(wash_r ~ Treatment + day + Treatment:day + (1|Colony) , data = dat_field1_long)

summary(m.5)

m.5.0 <- glmer.nb(wash_r ~ Treatment + day + (1|Colony) , data = dat_field1_long)

anova(m.5, m.5.0) # Not a significant interaction, though fairly close (P=0.15)

\# Analysis of change over time within treatment

emmeans(m.5, ~ day | Treatment, type = "response")

\# Looking at the estimates and considering the biological significance,

\# the "oxalic" group was more impressive than the "adjuvant" group, as in the previous analysis

\# BACI analysis based on mixed model passed through emmeans

emm.5 <- emmeans(m.5, ~ day * Treatment)

pairs(pairs(emm.5, simple = "day"), simple = "Treatment")

\# No significant differences in BACI analysis

\# Pre and post Varroa infestations - Year 2 ---------

dat_field2 <- read.csv("S6_Dataset.csv")

\# Look at normality

dat_field2 <- dat_field2 %>%

mutate(wash_delta = PostMitesPer100 - PreMitesPer100)

hist(dat_field2$wash_delta) # Graphically, this looks reasonably close to normally

\# distributed - perhaps because mite loads are closer to zero

shapiro.test(dat_field2$wash_delta)

\# Fails test for normality, though -

\# perhaps because of the high bump in middle

\# Mixed modeling

\# Data prep for mixed modeling

dat_field2_long <- dat_field2 %>%

rename(wash_final = PostMitesPer100,

wash_initial = PreMitesPer100

) %>%

pivot_longer(c(wash_final, wash_initial), names_to = c(".value", "day"), names_sep = "_") %>%

mutate(wash_r = round(100*wash), # Multiply by 100 and round this because technically negative binomial is supposed to be for count data

day = factor(day, levels = c("initial", "final"))

)

m.6 <- glmer.nb(wash ~ Treatment + day + Treatment:day + (1|Colony) , data = dat_field2_long)

summary(m.6)

m.6.0 <- glmer.nb(wash ~ Treatment + day + (1|Colony) , data = dat_field2_long)

anova(m.6, m.6.0) # Not a significant interaction, though fairly close (P=0.22)

\# Analysis of change over time within treatment

emmeans(m.6, ~ day | Treatment, type = "response")

\# Looking at the estimates and considering the biological significance,

\# the "oxalic" group was more impressive than the "adjuvant" group

\# BACI analysis based on mixed model passed through emmeans

emm.6 <- emmeans(m.6, ~ day * Treatment)

pairs(pairs(emm.6, simple = "day"), simple = "Treatment")

\# No significant differences in BACI analysis

\# Analysis of mite fall using repeated measures -----------------

dat_drop <- read.csv("S5_Dataset_edited_colnames.csv")

names(dat_drop)

dat_drop <- dat_drop %>%

mutate(drop_cumul = drop_0 + drop_2 + drop_4 + drop_7 + drop_14 + drop_21,

drop_0to4 = round(drop_0 + drop_2),

drop_0to6 = round(drop_0 + drop_2 + drop_4),

base = round(base),

drop_0 = round(drop_0),

drop_cumul = round(drop_cumul),

seams.x.wash = SeamsInitial*WashInitial

)

hist(dat_drop$drop_cumul, breaks = 10)

\# Negative binomial is what I would a priori go for, and it looks like a good distribution

m.7.norm <- lm(drop_cumul ~ Treatment + base, data = dat_drop)

hist(resid(m.7.norm), breaks = 10)

shapiro.test(resid(m.7.norm)) # Residuals are not statistically significantly non-normal

plot(m.7.norm) # As expected, the QQ plot is a little ugly

m.7 <- glm.nb(drop_cumul ~ Treatment + base, data = dat_drop)

summary(m.7)

m.7.0a <- glm.nb(drop_cumul ~ Treatment, data = dat_drop)

anova(m.7, m.7.0a) # Results: "baseline mite drop" was highly significant (P=0.010)

m.7.0b <- glm.nb(drop_cumul ~ base, data = dat_drop)

anova(m.7, m.7.0b) # Results: Treatment was highly significant (P<0.001)

\# Use emmeans to

\# a) make point estimates for "cumulative Varroa drop" for each treatment

emmeans(m.7, ~ Treatment, type = "response") # Confidence intervals also provided

\# These predictions are predicated on setting the baseline mite drop equal to the average baseline mite drop

\# b) Pairwise comparisons between treatments, Tukey-corrected

emmeans(m.7, pairwise ~ Treatment, type = "response")$contrasts

\# Results: The adjuvant group had 1.2 times as much cumulative mite drop,

\# though not statistically significant. This may still be worth reporting.

\# inspect model fit

plot(m.7) # Looks fine

\# Analyze just first X days

\# First 2 days

m.8 <- glm.nb(drop_0 ~ Treatment + base, data = dat_drop)

summary(m.8)

emmeans(m.8, ~ Treatment, type = "response")

emmeans(m.8, pairwise ~ Treatment, type = "response")$contrasts

\# 3.5 times as much drop in adjuvant as oxalic, and this is significant
