# Supplementary material for: Adjuvants to improve efficacy of miticides in managed honey bee (Apis mellifera) colonies to control Varroa destructor
Source: PLoS One. 2025 Jun 17;20(6):e0320037. doi: 10.1371/journal.pone.0320037 (PMC12173398; doi:10.1371/journal.pone.0320037)
Supplement: Attachment — Submitted filename: Response to Reviewers.docx [file pone.0320037.s009.docx]

Responses to Reviewer Comments

Manuscript ID PONE-D-25-07730 entitled “Adjuvants to improve efficacy of miticides in managed honey bee (*Apis mellifera*) colonies to control *Varroa destructor*”

Due: 07-May-2025

Brandon Shannon^1^*, Rui Zhang^2^, Lucas Marsh^1^, Reed M. Johnson^1^

^1^ Department of Entomology, The Ohio State University, Wooster, OH, United States of America

^2^ Department of Statistics, The Ohio State University, Columbus, OH, United States of America

* Corresponding author

Email: Shannon.325@OSU.edu

Thank you to the editor and reviewers for taking the time to review our manuscript and provide valuable feedback. We have modified the Funding Information, Competing Interested Statement, and Data Availability Statement to match the requirements of PLOS ONE. We have made modifications to our data analysis in response to Reviewer 2’s concerns. Please see responses to specific comments below.

Dear Dr. Shannon,

Thank you for submitting your manuscript to PLOS ONE. After careful consideration, we feel that it has merit but does not fully meet PLOS ONE’s publication criteria as it currently stands. Therefore, we invite you to submit a revised version of the manuscript that addresses the points raised during the review process.

Specifically, there are some concerns about the presentation expressed by both reviewers and more importantly, the second reviewer raises some concerns about the statistical models you used. Please either justify what you did or revise as suggested.

Please submit your revised manuscript by May 07 2025 11:59PM. If you will need more time than this to complete your revisions, please reply to this message or contact the journal office at [plosone@plos.org](mailto:plosone@plos.org). When you're ready to submit your revision, log on to [https://www.editorialmanager.com/pone/](https://urldefense.com/v3/__https:/www.editorialmanager.com/pone/__;!!KGKeukY!05clY3oYy9VZII8X0ywERfkaLD4WKXhbie78VydOqFRkDFgVlMtoa-U4DEnowUh_qmOK2jNlgaxe-mJsyR06$) and select the 'Submissions Needing Revision' folder to locate your manuscript file.

Please include the following items when submitting your revised manuscript:

- A rebuttal letter that responds to each point raised by the academic editor and reviewer(s). You should upload this letter as a separate file labeled 'Response to Reviewers'.
- A marked-up copy of your manuscript that highlights changes made to the original version. You should upload this as a separate file labeled 'Revised Manuscript with Track Changes'.
- An unmarked version of your revised paper without tracked changes. You should upload this as a separate file labeled 'Manuscript'.

If you would like to make changes to your financial disclosure, please include your updated statement in your cover letter. Guidelines for resubmitting your figure files are available below the reviewer comments at the end of this letter.

If applicable, we recommend that you deposit your laboratory protocols in protocols.io to enhance the reproducibility of your results. Protocols.io assigns your protocol its own identifier (DOI) so that it can be cited independently in the future. For instructions see: [https://journals.plos.org/plosone/s/submission-guidelines#loc-laboratory-protocols](https://urldefense.com/v3/__https:/track.editorialmanager.com/CL0/https:*2F*2Fjournals.plos.org*2Fplosone*2Fs*2Fsubmission-guidelines*23loc-laboratory-protocols/1/010f0195c64aba9d-fbfb0e70-433f-4c2e-89b6-67bcac0a0374-000000/iZ3BzTNobj5FmNHVyLwDfWTvTV15TFD28tcHHoeQAfM=203__;JSUlJSUl!!KGKeukY!05clY3oYy9VZII8X0ywERfkaLD4WKXhbie78VydOqFRkDFgVlMtoa-U4DEnowUh_qmOK2jNlgaxe-m9omLux$). Additionally, PLOS ONE offers an option for publishing peer-reviewed Lab Protocol articles, which describe protocols hosted on protocols.io. Read more information on sharing protocols at [https://plos.org/protocols?utm_medium=editorial-email&utm_source=authorletters&utm_campaign=protocols](https://urldefense.com/v3/__https:/track.editorialmanager.com/CL0/https:*2F*2Fplos.org*2Fprotocols*3Futm_medium=editorial-email*26utm_source=authorletters*26utm_campaign=protocols/1/010f0195c64aba9d-fbfb0e70-433f-4c2e-89b6-67bcac0a0374-000000/R6j9zBwrFGklmi5FVlrR1BhIgxE3VVvLKaYJLF_EkHc=203__;JSUlJSUl!!KGKeukY!05clY3oYy9VZII8X0ywERfkaLD4WKXhbie78VydOqFRkDFgVlMtoa-U4DEnowUh_qmOK2jNlgaxe-s6HgPcb$).

We look forward to receiving your revised manuscript.

Kind regards,

Olav Rueppell
Academic Editor
PLOS ONE

Journal Requirements:

When submitting your revision, we need you to address these additional requirements.

1. Please ensure that your manuscript meets PLOS ONE's style requirements, including those for file naming. The PLOS ONE style templates can be found at [https://journals.plos.org/plosone/s/file?id=wjVg/PLOSOne_formatting_sample_main_body.pdf](https://urldefense.com/v3/__https:/journals.plos.org/plosone/s/file?id=wjVg*PLOSOne_formatting_sample_main_body.pdf__;Lw!!KGKeukY!05clY3oYy9VZII8X0ywERfkaLD4WKXhbie78VydOqFRkDFgVlMtoa-U4DEnowUh_qmOK2jNlgaxe-kEYW8jm$) and [https://journals.plos.org/plosone/s/file?id=ba62/PLOSOne_formatting_sample_title_authors_affiliations.pdf](https://urldefense.com/v3/__https:/journals.plos.org/plosone/s/file?id=ba62*PLOSOne_formatting_sample_title_authors_affiliations.pdf__;Lw!!KGKeukY!05clY3oYy9VZII8X0ywERfkaLD4WKXhbie78VydOqFRkDFgVlMtoa-U4DEnowUh_qmOK2jNlgaxe-o2Pie5T$)

Thank you, we have ensured that our file names match the requirements for PLOS ONE.

2. We note that the grant information you provided in the ‘Funding Information’ and ‘Financial Disclosure’ sections do not match.

When you resubmit, please ensure that you provide the correct grant numbers for the awards you received for your study in the ‘Funding Information’ section.

Thank you, we have ensured that the grant numbers in the ‘Funding Information’ section of the manuscript submission are correct.

3. Thank you for stating the following financial disclosure:
We thank the National Honey Board with Project Apis m., the California State Beekeeper’s Association, The Ohio State University CFAES Internal Grants Program, USDA-NIFA-SCRI (2023-51181-41246) and state and federal appropriations to The Ohio State University College of Food, Agriculture and Environmental Science (OHO01558-MRF).

Please state what role the funders took in the study. If the funders had no role, please state: "The funders had no role in study design, data collection and analysis, decision to publish, or preparation of the manuscript."
If this statement is not correct you must amend it as needed.
Please include this amended Role of Funder statement in your cover letter; we will change the online submission form on your behalf.

 Thank you, we have added this statement to the manuscript and included the full revised statement in the Cover Letter.

4. Thank you for stating the following in the Competing Interests section:
I have read the journal's policy and the authors of this manuscript have the following competing interests: A patent application (PCT/US2024/056156; ADJUVANTS TO IMPROVE EFFICACY OF VARROA CONTROL ACTIVE INGREDIENTS IN MANAGED HONEY BEE COLONIES) was filed on November 15, 2024, by the Ohio State Innovation Foundation with inventors Reed Johnson and Brandon Shannon.

Please confirm that this does not alter your adherence to all PLOS ONE policies on sharing data and materials, by including the following statement: "This does not alter our adherence to PLOS ONE policies on sharing data and materials.” (as detailed online in our guide for authors [http://journals.plos.org/plosone/s/competing-interests](https://urldefense.com/v3/__http:/journals.plos.org/plosone/s/competing-interests__;!!KGKeukY!05clY3oYy9VZII8X0ywERfkaLD4WKXhbie78VydOqFRkDFgVlMtoa-U4DEnowUh_qmOK2jNlgaxe-ijlbNN3$)). If there are restrictions on sharing of data and/or materials, please state these. Please note that we cannot proceed with consideration of your article until this information has been declared.

Please include your updated Competing Interests statement in your cover letter; we will change the online submission form on your behalf.

 Thank you, we have added this statement to the manuscript and included the full revised statement in the Cover Letter.

5. Please provide a complete Data Availability Statement in the submission form, ensuring you include all necessary access information or a reason for why you are unable to make your data freely accessible. If your research concerns only data provided within your submission, please write "All data are in the manuscript and/or supporting information files" as your Data Availability Statement.

Thank you, we have added a Data Availability Statement in the manuscript submission: “All data can be found in the manuscript and/or supporting information files. Code used for statistical analysis can be found in the supporting information files.”

6. We are unable to open your Supporting Information file [S2_File.R]. Please kindly revise as necessary and re-upload.

Thank you, we have revised S2 as a text file instead of an R script file.

7. Please review your reference list to ensure that it is complete and correct. If you have cited papers that have been retracted, please include the rationale for doing so in the manuscript text, or remove these references and replace them with relevant current references. Any changes to the reference list should be mentioned in the rebuttal letter that accompanies your revised manuscript. If you need to cite a retracted article, indicate the article’s retracted status in the References list and also include a citation and full reference for the retraction notice.
Thank you, we have reviewed our references. They are correct to the best of our knowledge.

Reviewers' comments:

Reviewer's Responses to Questions

**Comments to the Author**

1. Is the manuscript technically sound, and do the data support the conclusions?

The manuscript must describe a technically sound piece of scientific research with data that supports the conclusions. Experiments must have been conducted rigorously, with appropriate controls, replication, and sample sizes. The conclusions must be drawn appropriately based on the data presented.

Reviewer #1: Yes

Reviewer #2: Partly

2. Has the statistical analysis been performed appropriately and rigorously?

Reviewer #1: I Don't Know

Reviewer #2: No

3. Have the authors made all data underlying the findings in their manuscript fully available?

The [PLOS Data policy](https://urldefense.com/v3/__http:/track.editorialmanager.com/CL0/http:*2F*2Fwww.plosone.org*2Fstatic*2Fpolicies.action*23sharing/1/010f0195c64aba9d-fbfb0e70-433f-4c2e-89b6-67bcac0a0374-000000/zQkugk7DkOhJirmkdb88MHTzLpju2C6S-YJCOcnHDe8=203__;JSUlJSU!!KGKeukY!05clY3oYy9VZII8X0ywERfkaLD4WKXhbie78VydOqFRkDFgVlMtoa-U4DEnowUh_qmOK2jNlgaxe-leK8mFw$) requires authors to make all data underlying the findings described in their manuscript fully available without restriction, with rare exception (please refer to the Data Availability Statement in the manuscript PDF file). The data should be provided as part of the manuscript or its supporting information, or deposited to a public repository. For example, in addition to summary statistics, the data points behind means, medians and variance measures should be available. If there are restrictions on publicly sharing data—e.g. participant privacy or use of data from a third party—those must be specified.

Reviewer #1: Yes

Reviewer #2: Yes

4. Is the manuscript presented in an intelligible fashion and written in standard English?

PLOS ONE does not copyedit accepted manuscripts, so the language in submitted articles must be clear, correct, and unambiguous. Any typographical or grammatical errors should be corrected at revision, so please note any specific errors here.

Reviewer #1: Yes

Reviewer #2: Yes

5. Review Comments to the Author

Please use the space provided to explain your answers to the questions above. You may also include additional comments for the author, including concerns about dual publication, research ethics, or publication ethics. (Please upload your review as an attachment if it exceeds 20,000 characters)

**Reviewer #1: General comments:**
This study by Shannon et al. investigated the efficacy of oxalic acid and other acaricides in glycerin formulations combined with several adjuvants for the control of the parasitic mite Varroa destructor. A question of interest was if adjuvants increase the varroacidal efficacy of acaricides. Laboratory cage assays were used to screen the acaricidal effect of three products in combination with several adjuvants. One acaricide (oxalic acid) and one adjuvant (Ecostep BC-12) were selected for field testing. Results indicate that the adjuvant increased the efficacy of oxalic acid against Varroa, but the acaricide was ineffective relative to the control, when used alone. The manuscript is well written and in my opinion only requires a few minor editions and reorganization for improvement that I mention under specific comments.

Thank you to Reviewer 1 for taking the time to review our manuscript and provide constructive feedback.

**Specific comments:**
1. Titles of sections (Abstract, Methods, Results, etc.) are typed in red font. Is this a requirement of the journal? If so, leave it as it is, if not, please follow the instructions for authors of the journal.

Font color has been changed to black to match journal formatting guidelines.

2. Abstract, L23. “soaked strips; field trials evaluated oxalic acid combined with..” I suggest using a period instead of a semicolon to separate lab trials from field trials. Also, please mention why oxalic acid and Ecostep BC-12 were selected for field trials.

Thank you, this has been revised.

“in glycerin-soaked strips. Field trials evaluated the best performing active ingredient-adjuvant combination from cage trials, oxalic acid combined with Ecostep BC-12^®^ adjuvant in glycerin-soaked strips.”

3. Abstract. Please provide some numerical and statistical comparisons from your results between the treatment with the adjuvant and the treatment without the adjuvant.

Please see the following revisions:

“Field trials with oxalic acid and adjuvant in glycerin caused a significant decrease of 3.1 (SE: 2.1 – 4.1) *Varroa* per 100 bees in year 1 and a significant reduction in *Varroa*, relative to the solvent control, from 2.1 to 0.4 mites per 100 bees, in year 2.”

4. Methods, Treatments, L216-28. Please mention somewhere in Methods why oxalic acid and Ecostep BC-12 were selected for field trials.

Please see the following revisions to the methods section:

L226: “The combination of oxalic acid active ingredient with Ecostep BC-12® adjuvant was chosen for field trials as it had the highest efficacy in cage trials.”

5. Data Analysis L233-239. Differences in Varroa levels were either analyzed with t tests or ANOVAs. Were these data checked for normality and homoscedasticity before analyzing them? If so, what tests did you use? Please mention them.

Thank you for pointing this out. Our statistical methods have been changed to using a generalized linear mixed model (GLMM) to determine pre- and post-treatment differences. Please see text excerpt for description of revised statistical methods:

L246: “Efficacy within treatments was compared by modelling individual wash counts using a generalized linear mixed model (GLMM) with a negative binomial distribution and a random intercept for colony (73).”

6. Discussion. I suggest you to start with a small paragraph outlining your main results, both, from cages and from hives. Following paragraphs should discuss your results and conclusions in comparison with other studies previously published.

Thank you, we have moved the start of the conclusion that describes the main results to the start of the discussion.

7. Discussion first and second paragraphs (L 330-349) are justifications of methods used. I suggest shortening the text containing this information and move the text to the Methods section.

Thank you, this has been shortened and moved to the methods section.

8. Discussion L 371-374. Again, the arguments presented here are a justification to conduct the study and thus they belong in the Introduction section.

Thank you, this has been moved to the introduction.

9. Conclusion. I suggest first mentioning results and conclusions from your cage experiments before mentioning those of your field trial experiments.

This is a great suggestion, we have moved the first half of the conclusion paragraph to the beginning of the discussion and removed the section header “Conclusion”.

10. Conclusion L 406: “oxalic alone” Do you mean “oxalic acid alone”?

Thank you, this has been corrected.

**Reviewer #2:**

## Overall

This seems to be a well-conducted study. Appropriate methods are used for the LC50 analysis. A few more key details are needed: sample size for cage trials (number of cages per treatment), and initial and ending mite levels for the field experiment.

I have some suggestions (in blue text) for alternative statistical approaches for some the analyses, mostly to incorporate the “generalized” version of the linear modeling. I think reanalyses will let you say more about the effect of adjuvants in cage trials, but likely you will need to pull back some of your statements about significant effects in the field trial.

I appreciate you including your data, as this allowed me to test some alternative approaches. At the end of this document I have included the R code I used when reviewing your analyses. If you paste this into an R script, some of this code may be helpful, or at least will let you follow my process of reviewing your stats.

We thank Reviewer 2 for taking the time to review our manuscript and provide constructive feedback. We are grateful for your contributions and suggestions for the statistical analysis.

## Abstract

- Nice and clear

## Introduction

- L38-39. Remove the word “average” to reflect the kind of estimate that’s made, an estimate of the loss rate for the population of honey bee colonies. For the CAPA survey please confirm, but you will probably remove “average” here as well.

Thank you for catching this, “average” has been removed for reference to both surveys.

- L41-42. This is a little different than what you have written. 60% of commercial beekeeping operations reported it as a leading cause of winter loss.

Thank you, this has been corrected.

- L42-43. This list of pathogens may not be necessary since “associated diseases” was already mentioned.

This sentence has been removed.

- L67. Spelling of Mitchondrial

Thank you, this has been corrected.

- L84. Reconsider word choice of “greaten”

Changed to “increase”

- L93. Capitalization and italicization of “Varroa”

Thank you, this has been corrected.

## Materials and Methods

- Data analysis methods for the LC50 determination should be briefly added. Reading the manuscript I think you used the drc package to fit 2-parameter log-logistic dose-response models.

Please see the following added sentence describing data analysis:

L123: “Data analysis was performed with the drc package in R (52) using 2-parameter log-logistic dose-response models, as performed by (45).”

45. Shannon B, Walker E, Johnson RM. Toxicity of spray adjuvants and tank mix combinations used in almond orchards to adult honey bees (Apis mellifera). J Econ Entomol. 2023 Oct 1;116(5):1467–80.
52. Ritz C, Baty F, Streibig JC, Gerhard D. Dose-Response Analysis Using R. PLOS ONE. 2015 Dec 30;10(12):e0146021.

- L97. Do you have an analysis of the chemical constituents of the clove oil used? Or if not, any reference for usual composition and / or how much these vary?
- L99. How is 102.5% purity possible? Spelling of Scientific

Spelling corrected. 102.5% purity is not an error; this is the specification from Thermo Fisher Scientific. It is likely that purity above 100% is possible because oxalic acid that is not fully hydrated would have a greater relative concentration of oxalic acid compared to pure oxalic acid dihydrate. I have listed below the COA for our most recent lot of Oxalic Acid Dihydrate, which has specifications for 99.5 – 102.5%.

<https://www.fishersci.com/us/en/catalog/search/cofa/getcert?catalognumber=A219&lotnumber=242439&docType=01>

- L127 and L134-136. Only one of these statements is needed.

Thank you for catching this repeated statement, the statement at line 127 has been removed.

- L133. “Sonicating” or rephrase

Corrected

- L152. Were the weigh boats or trays under the cages covered with Vaseline as in the Rinkevich protocol? I am only asking since it is not mentioned here or on L180

We had only mentioned deviations from the Rinkevich protocol. We added the following sentence to reduce confusion.

L160: “The design of cages above petroleum jelly-coated weigh boats was modified from Rinkevich, 2020 (17) (S1 Text, Fig A).”

- L170. Replace “phoretic mites” with “mites in the dispersal phase” or “mites on adult bees” as phoresy refers to non-feeding transport.

Thank you, this has been corrected

- L176. The description of cage treatment series could be more specific. “Four active ingredient-adjuvant combinations” is ambiguous and could be a concentration range of one adjuvant, different adjuvants, or both. This could be your place to describe the blocking to limit confounding effects. Sample size is needed here.

Thank you for pointing this out. We have improved the text to the following:

L177: “Each cage treatment series consisted of 6 treatments: a negative control (glycerin solvent only), active ingredient control (active ingredient in glycerin), and four combinations of that same active ingredient in glycerin with four different adjuvants. Using a cage assay rather than a traditional topical application or glass vial application to Varroa better simulates the exposure that mites would face in a colony environment, but may introduce additional sources of variability compared to other topical application methods (59), from variable age (60–62), Varroa loads (59), bee health (12,63,64), and colony strength (65). This variability was managed by performing each set of treatments, assigned in random order, with the same cohort of shaken bees. A minimum of 3 replicates were performed for each treatment.”

- L187. Kruskal-Wallace test for differences in bee and Varroa mortality in cages
  - Why not run logistic regression instead of doing a nonparametric test?
  - I tried this on the clove data and found significant differences between treatments. Logistic regression seems to give you more power to detect differences.
  - Code provided at end of comments.
  - Please let me know if you see any issues with this approach

We agree that logistic regression is a better approach. Thank you for taking the time to perform the analysis and share your code with us. We have used your code to perform logistic regression for both the *Varroa* and honey bee mortality in the cage trial. The Results section text, Fig 1, S1_Text Table A, and S2_File have been updated to show the revised interpretation and code from the revised statistical analysis. Please see the below text revisions:

Methods:

L196: “A logistic regression was used to determine significant differences in bee mortality and Varroa control efficacy (68), followed by a Tukey HSD post-hoc test (69).”

Results:

L268: “*Clove oil*

There was a significant difference in 24-hour bee mortality among treatments (logistic regression, P = 0.028; S1 Text, Table A; S4 Dataset). Tukey’s post-hoc tests determined that Ecostep BC-12 with clove oil caused significantly more bee mortality than the glycerin control (P = 0.050). A significant difference was observed in 24-hour *Varroa* efficacy (logistic regression, P < 0.001; Fig 1A; S1 Text, Table A; S4 Dataset). Tukey’s post-hoc correction determined that Ecostep AE-13®, Ecostep BC-12®, Ecostep SE-11®, and Silwet L-7500 Copolymer® with clove oil caused significantly increased *Varroa* efficacy (P < 0.05) compared to the glycerin control, and that Ecostep AE-13®, Ecostep BC-12®, and Silwet L-7500 Copolymer® with clove oil caused significantly increased *Varroa* efficacy (P < 0.05) compared to the clove oil alone.

*Fenpyroximate*

There was a significant difference in 24-hour bee mortality among treatments (logistic regression, P < 0.001; S1 Text, Table A; S4 Dataset). Pairwise Tukey’s post-hoc tests determined significantly increased bee mortality (P < 0.05) for Ecostep AE-13®, Ecostep BC-12®, and Silwet L-7500 Copolymer® with fenpyroximate compared to the glycerin control. A significant difference was observed in 24-hour *Varroa* efficacy (logistic regression, P < 0.001; Fig 1B; S1 Text, Table A; S4 Dataset). Tukey’s post-hoc tests determined that fenpyroximate alone, as well as Ecostep BC-12®, Ecostep CE-13®, Ecostep SE-11®, and Silwet L-7500 Copolymer® with fenpyroximate caused significantly increased *Varroa* efficacy (P < 0.05) compared to the glycerin control, and that Ecostep BC-12®, Ecostep CE-13®, Ecostep SE-11®, and Silwet L-7500 Copolymer® with fenpyroximate caused significantly increased *Varroa* efficacy (P < 0.05) compared to the fenpyroximate alone.

*Oxalic acid*

There was a significant difference in 24-hour bee mortality among treatments (logistic regression, P = 0.018; S1 Text, Table A; S4 Dataset). However, pairwise Tukey’s post-hoc tests determined no significant differences in bee mortality between any of the treatments. A significant difference was observed in 24-hour *Varroa* efficacy (logistic regression, P < 0.001; Fig 1C; S1 Text, Table A; S4 Dataset). Tukey’s post-hoc tests determined that oxalic acid alone, as well as Ecostep BC-12®, Ecostep CE-13®, Ecostep SE-11®, and Silwet L-7500 Copolymer® with oxalic acid caused significantly increased *Varroa* efficacy (P < 0.05) compared to the glycerin control, and that only Ecostep BC-12® caused significantly increased *Varroa* efficacy (P < 0.05) compared to the oxalic acid alone. The combination of oxalic acid with Ecostep BC-12® was the most efficacious treatment of all three treatment series, with an efficacy of 96.4% (standard deviation = 5.0).”

Additionally, Fig 1 and its caption has been revised to demonstrate the new statistical methods:

L280: “Efficacy of 3% Clove Oil (A), 0.2% Fenpyroximate (B), and 20% Oxalic Acid (C) treatments in cage trials, where efficacy is determined to be the number of *Varroa* mites that fell in 24 hours divided by the total number of *Varroa* mites in each cage. Adjuvant concentrations were 0.5% for all Ecostep adjuvants and 0.1% for Silwet L-7500 copolymer. Significant difference (P < 0.05) from the glycerin control is indicated by an asterisk (*) and significant difference (P < 0.05) from the active ingredient alone is indicated by a dagger (†).”

- L188. “Wilcoxon”

Removed since this was revised to logistic regression.

- L198-199. Great that you stratified. Great that you described the spatial arrangement of treatments. For completeness, specify whether the stratification was based on pre-treatment alcohol wash or sticky board mite fall.

Clarified that stratification was based on pre-treatment alcohol wash

- L213. “manila”

Corrected

- L213. Closed or open? Perhaps specify dimensions as you do for the complete Swedish sponge.

Thank you, we have clarified that folders placed under colonies were open and then were closed when removed.

L220: “Mite drop was monitored by placing open letter-sized (43.2 cm x 27.9 cm) manila file folders (Staples, Framingham, MA, USA) coated with Vaseline® petroleum jelly (Unilever, Egewood Cliffs, NJ, USA) under screened bottom boards. After folders were removed, they were folded closed and frozen at -20º C for a minimum of 24 hours and stored under these conditions until counted.”

- L235-236. I looked into the paired t test.
  - For year 1 the assumption of normally distributed deltas is violated, so the t test is not appropriate. I recommend generalized linear modeling (GLM) using a negative binomial distribution
  - The GLM I tried did not support a treatment x time interaction. This could be further finessed by accounting for both the bee number and the varroa number per sample. Code for my quick analysis, and a finessed analysis are provided at end of comments.
  - Unless a more finessed analysis gives different results, this calls for the removal of statements like on Line 24-25 “oxalic acid and adjuvant in glycerin caused a significant decrease in Varroa in Year 1”. This statement also highlights the OA-adjuvant effect over the OA effect even though OA had a better estimated effect, but more variation. This highlights statistical significance over biological significance, which can lead to a bottom-line conclusion that is not supported. Readers may think the adjuvant increased performance in year 1.
  - For year 2, the paired t test may be OK as the normality of deltas looks better, graphically. That said, the values fail a normality test, so the significant difference between control and OA-adjuvant should be re-evaluated as well.
  - On a different note, Figure S1B does a great job of showing patterns in the data, which are suggestive of a benefit of the adjuvant, even if not statistically significant.

Thank you for your detailed review of our statistical methods. We have revised the statistical methods to perform a GLMM instead of a t-test. The results, figure, and discussion have been revised to match the new statistical analysis. All R code for statistical analysis is now included in supplemental information S2. Please see the below revised text:

Methods:

L245: “Field trial results were analyzed independently for both years, using the same statistical analysis methods. Efficacy within treatments was compared by modelling individual wash counts using a generalized linear mixed model (GLMM) with a negative binomial distribution and a random intercept for colony (73).”

Results:

L318: “In year 1, the glycerin control colonies had a 1.6-fold increase in *Varroa* per 100 bees (Fig 2A; S1 Text, Table B, Fig B; S5 Dataset), an average increase of 8.4 *Varroa* per 100 bees (95% CI = 1.7, 15.1), from 12.4 (SD = 7.1) to 20.8 (SD = 16.3). However, this increase was not statistically significant (z = 1.203, P > 0.1). The oxalic acid alone had a 43.4% reduction in *Varroa* per 100 bees, which was an average decrease of 4.3 *Varroa* per 100 bees (95% CI = -8.7, 0.1), from 12.1 (SD = 7.6) to 7.6 (SD = 9.6). This decrease was not statistically significant (z = -1.576, P > 0.1). A 35.2% reduction in *Varroa* per 100 bees was observed between the pre- and post-treatment ethanol washes for the oxalic acid plus adjuvant treatment, which was an average decrease of 3.1 *Varroa* per 100 bees (95% CI = -4.1, -2.1), from 10.9 (SD = 7.1) to 7.8 (SD = 7.5). This decrease was also not statistically significant (z = -1.155, P > 0.1).

In year 2, a 2.6-fold increase in *Varroa* per 100 bees was observed for colonies in the glycerin control (Fig 2B; S1 Text, Table B, Fig B; S5 Dataset), an average increase of 1.3 *Varroa* per 100 bees (95% CI = 0.9, 1.7), from 0.8 (SD = 0.8) to 2.1 (SD = 0.9). This increase was statistically significant (z = 22.020, p < 0.0001). In the oxalic acid alone treatment, *Varroa* per 100 bees increased by 2.8-fold after treatment, an average increase of 0.7 *Varroa* per 100 bees (95% CI = 0.5, 0.8), from 0.4 (SD = 0.5) to 1.0 (SD = 0.5). This increase was statistically significant (z = 16.102, p < 0.0001). In the oxalic acid plus adjuvant treatment, a 29% reduction in *Varroa* per 100 bees was observed between the pre- and post-treatment ethanol washes, which was an average decrease of 0.2 *Varroa* per 100 bees (95% CI = -0.5, 0.2), from 0.6 (SD = 0.7) to 0.4 (SD = 0.4). This decrease was statistically significant (z = -5.016, P < 0.0001).”

Figure:

L329: “Fig 2. *Varroa* levels from pre- and post-treatment alcohol washes in year 1 (A) and year 2 (B) field trials. Points indicate mites per 100 bees for each colony, and lines connect pre- and post-treatment data for each individual colony. A statistically significant difference (P < 0.05) between post- and pre-treatment *Varroa* levels within treatments, determined using a GLMM, is indicated with an asterisk (*).”

- L239-240. Mixed modeling procedure
  - I am glad you accounted for the baseline mite drop. Is there a good theoretical rationale for including all the predictors? Maybe that is all you need as a covariate (see my code). Check for collinearity, for example by testing the model with `vif()` from the `car`package.
  - As our experience is that mite drop is usually not appropriately modeled with a normal distribution, consider a generalized procedure. I again recommend starting with a negative binomial distribution. That said, when modeled with a normal distribution, the residuals were not significantly non-normal, though by eye the histogram did not look great. You could make an argument that this is a case where the data allow the assumption of normality.

Thank you for your comments on the statistical methods. We have revised our methods to use a negative binomial generalized linear model to account for overdispersion in count data. We began with a base model including only Treatment and used forward selection to evaluate the added value of additional predictors: Location, SeamsInitial,wash_initial, and baseline mite drop. The final model included all predictors. And diagnostics support the adequacy of the model for inference. The R code for the data analysis is included in S2. Please see the revised text:

Methods:

L248: “To analyze cumulative mite drop over the full 23-day treatment period in year 1, we modeled the data using a negative binomial generalized linear model (74) to account for overdispersion in count data (S2 Text). The model included treatment and used forward selection to choose variables for inclusion in the model, which included the following predictors: apiary, initial seam counts, initial mite wash, and baseline mite drop. A second negative binomial generalized linear model with the same predictors was used for analyzing cumulative mite drop counts over the first 2 and 4 days of treatment.”

Results:

L346: “Over the 23-day treatment period in year 1, colonies treated with oxalic acid with adjuvant had a 4.7-fold increase in cumulative mite drop compared to the glycerin control (p < 0.001; Table 3; Fig 3; S5 Dataset). In the first 4 days, colonies treated with oxalic acid with adjuvant had a 6.4-fold increase in total mite drop compared to the glycerin control (p < 0.001), and a 1.46-fold increased total mite drop compared to oxalic acid alone, but this difference was not statistically significant (p = 0.117). In the first 2 days, colonies treated with adjuvant had an 8.5-fold increase in mite drop compared to the glycerin control (P < 0.001), and a 2.8-fold increase in mite drop compared to oxalic acid alone, which was statistically significant (P < 0.001).”

## Results

- Initial (and preferably ending) Varroa infestations are needed for context.

Thank you, we have added initial and final *Varroa* per 100 bees in addition to the difference between the two for years 1 and 2.

- L248. “was”

Corrected

- L260. Move figure reference. Fig 1A refers to efficacy against Varroa, not effects on bee mortality. Check the other references in the statement.

Thank you, this has been corrected

- L266. Move figure reference. Fig 1A refers to efficacy against Varroa, not effects on bee mortality. Check the other references in the statement.

Thank you, this has been corrected

- L269-270. Move figure reference. Fig 1A refers to efficacy against Varroa, not effects on bee mortality. Check the other references in the statement.

Thank you for the above 3 corrections, all figure references have been amended to statements only about *Varroa*.

- L283-290. By presenting only deltas, we lack important context. How high were pre-and post-treatment mite levels in the alcohol wash for both years?

Thank you, pre- and post-treatment mite levels were added

- Figure 2. I think it would be more informative if you replace Figure 2 (showing the deltas) with Figure S1B. I think Figure S1B is excellent. You could remove Figure 2 or move it to the supplemental materials.
  - If you take my earlier suggestion to remove mentions of “significant decrease of Varroa per 100 bees in adjuvant group for year 1” this would necessitate removing the dagger in Pane A of Figure S1B
  - For the Figure S1B legend, it should say “a significant difference between treatments” Thank you, this has been corrected.
  - Additionally, you may need to remove significance between treatments

Thank you for this suggestion. We have swapped Figure 2 and Figure S1B so that the figure that demonstrates the pre- and post-treatment levels is the one that is included in the manuscript. We have modified the caption and symbols on the figure to match the revised statistical analysis.

- Figure 3. Add baseline mite fall measurements.

We have added baseline mite fall measurements and changed the measurements to cumulative measures rather than non-cumulative, since the negative binomial GLM uses cumulative data for analysis. The original figure (with baseline measurement added) was added into supplemental information so that we could keep the reference in the discussion.

- L301. The units (“Varroa per 100 bees”) are best placed with the number, i.e., following “1.3”

Thank you, this has been corrected

- L313. Results for analysis of mite fall. This usually needs to be a generalized model, not just a mixed model – initial suggestion: negative binomial distribution.

Thank you for this suggestion. A negative binomial GLM is now being used. See earlier comment for changes to text and figures. All statistical analysis is available in supplemental information S2.

## Discussion / Conclusion

- The Discussion could be made more focused by reducing limitations, literature gaps, and future steps.

Thank you, we have reduced some of the limitations and moved the rationale to the appropriate sections.

- I think the Discussion should be more focused on answering the “aim of the research” statement at the end of the Introduction. What did your research reveal about the usefulness of adjuvants for Varroa control? What uncertainties remain? Do you have any speculations about the impact of variables that changed between the two years of the field study? Any further explanation for why the oxalic-adjuvant combination was pursued and not the other active ingredients. Your suggestions for “next steps forward” are good, but they will be stronger if you can focus on how results from this study indicate which next steps should be pursued. With so many variables that can be manipulated, perhaps you have ideas for how to approach this systematically.

Thank you for your suggestion. We have focused more on how the adjuvant improved efficacy of oxalic acid in year 2, while oxalic acid alone was ineffective. We have also focused more on the increased speed of action for the adjuvant treatment compared to oxalic acid alone over the first 2 days of treatment. We feel that addressing the main aim of the paper, whether adjuvants can improve efficacy of miticides, strengthens the other arguments in the discussion.

- Your Discussion may benefit from subheadings if the journal style allows.

After the suggested revisions, we think that subheadings are no longer necessary, since the discussion is more focused and concise.

- In the current form, I don’t think having a separate Conclusions section is helpful. The content from the Conclusion would be better at the start of the Discussion.

Thank you, we have moved the conclusion content to start the discussion and removed the conclusion section header.

- L401-410. This content would be good to start your Discussion with.

Thank you for the two above suggestions. We have moved the start of the Conclusions section to the beginning of the discussion, and removed the separate conclusions section.

- L330-343. This is a long discussion of limitations. It is clear that you had appropriate procedures in place to address them. Since you blocked all your cage trial treatments within cohorts of shaken bees, this section seems unnecessary here but could briefly be added to the Materials and Methods.

Section was shortened and moved to materials and methods.

- L344-349. I think this can also be removed. The main thing is that you used the same method for all groups and timepoints that you compared. This point is made in the BEEBOOK Varroa standard methods paper (Dietemann et al. 2013, Section 4.2.3.1.2.4. Assessing the efficiency of dislodging method), so you could cite this in your Methods. There you may want to simply disclose that you did not fully evaluate the recovery efficacy of your alcohol wash but state that the same methods were used at all time points.

Thank you, this section was removed and a statement was added to the methods, with the Dietemann et al., 2013 citation. Additionally, the citation for Fries et al., 1991, was added as the original citation from the Dietemann et al. book.

- L350-360. This is the first part that truly seems to be discussing your results. Keep this.

Thank you for the feedback.

- L361-370. These are good Discussion points. They would be better if they were more connected to your results.

Thank you. The following have been added to connect these to our results:

L390: “The decreased mite drop observed between the collection periods of days 7-9 and days 14-16 may indicate that oxalic acid treatments may become less effective over time (Table 3; S1 Text, Fig C).”

- L371-374. This belongs in the Introduction, or can be removed.

Thank you, this has been moved to the introduction.

- L375-380. This could be part of a “next steps” paragraph

Thank you, this has been removed from this paragraph and put with the next steps

- 381-399. As written, this belongs in an Introduction.

This has been revised to more specifically relate the results of this study to possible mechanisms.

L405: “The increased mite drop with the combination of oxalic acid and adjuvant that was observed over the first two days may be the result of increased spreading throughout the colony. As all five adjuvants used in this study are listed by the manufacturer as surfactants, they may improve the efficacy of the miticide through increased spreading on the honey bees or the *Varroa* themselves, thereby increasing the exposure of *Varroa* to the miticide. Increased spreading of the miticide throughout the colony and over the bee cuticle is critical in mediating exposure of *Varroa* to the miticide, as most *Varroa* on adult bees are located under the honey bee abdominal sternites (85). In addition to improving spreading, adjuvants may also act through mechanisms that improve spiracular or cuticular penetration of the miticide in *Varroa*, as demonstrated in other arthropod pests (50,86–89). It is also possible that adjuvants may have intrinsic toxicity to *Varroa*, as has been observed in spider mites (89–92), aphids (92–95), thrips (96), cockroaches (97), and mosquitoes (43,98). However, the adjuvants used in this study were not tested on their own for toxicity to *Varroa*. Other adjuvants not used in this study are toxic to honey bees (45,99–104) and other bee species (105,106), so it is important to perform preliminary testing prior to including other adjuvants in candidate miticide formulations. The mechanism of action for adjuvant toxicity to arthropods is not well understood, but it is thought that adjuvants act similarly to insecticidal soaps (45,91,107,108), which can disrupt the arthropod cuticle, break down cell membranes, and reduce water surface tension to cause spiracular drowning (98,109).”

Funding

- L426-427. There was a recent notice that USDA-NIFA funding should be acknowledged in a specific format: <https://content.govdelivery.com/accounts/USDANIFA/bulletins/3d43d38>

Thank you, the format has been corrected.

## Code used when reviewing analyses

Thank you for providing this code. It was very helpful in revising the statistical analysis.

library(MASS)

library(tidyverse)

library(lme4)

library(emmeans)

library(car)

\# Logistic regression to assess differences in Varroa mortality in cages ----------

\# As a demo, run this on the clove oil subset of data

\# data import

dat_cage <- read.csv("S4_Dataset.csv")

\# data prep

dat_cage_clove <- dat_cage %>%

filter(AI == "Clove Oil") # Omits GlyOnly controls, I need to approach data prep differently

\# second try to filter the data that are relevant to the clove subset of data

dat_cage_clove <- dat_cage[1:31,]

dat_cage_clove <- dat_cage_clove %>%

mutate(

PFA = case_when(AI == "Control" ~ "GlyControl", .default = PFA)

)

\# syntax - cbind(successes, failures) as a function of [predictor] or [intercept]

m.1 <- glm(cbind(X24h.varroa.dead, Varroa.Wash) ~ PFA, family = binomial, data = dat_cage_clove)

m.1.0 <- glm(cbind(X24h.varroa.dead, Varroa.Wash) ~ 1, family = binomial, data = dat_cage_clove)

anova(m.1, m.1.0)

\# Result: significant effect of treatment (P=3.386e-15)

summary(m.1)

\# Use emmeans to

\# a) make point estimates for "Varroa mortality probability" for each treatment

emmeans(m.1, ~ PFA, type = "response") # Confidence intervals also provided

\# b) Pairwise comparisons between treatments, Tukey-corrected

emmeans(m.1, pairwise ~ PFA, type = "response")$contrasts

\# here we are seeing significant differences between treatments

\# Pre and post Varroa infestations - potential finessed approach ---------

\# Some reorganizing of original data would be necessary to try this

```
## A model setup you could try -
## The response variable is the number of Varroa in the final sample (integer).
## There is an offset for the "denominator" of these samples,
## i.e., the number of bees in the final samples (may need to be integer).
## This is transformed by the link function (log),
## The covariate is the initial percent varroa infestation (can be decimal)
## Finessed model
```

\# m <- glm.nb(num_varroa_final ~ Treatment + percent_varroa_initial +

\#	offset(log(num_bees_final)), data=dat)

```
## To get emmeans predictions from finessed model you need to include what value
## of the offset you want to make predictions on. This would usually be
## mites per 100 bees, by setting offset = log(100)
```

\# preds <- emmip(m, ~ Treatment, type = "response", CIs = TRUE, plotit = FALSE, offset = log(100))

\# Pre and post Varroa infestations - Year 1 ---------

\# Demo on S5, Year 1 field trial dataset

\# Description: paired t test

dat_field1 <- read.csv("S5_Dataset.csv")

\# data prep

dat_field1_sub_control <- dat_field1 %>%

filter(Treatment == "Control")

dat_field1_sub_oxalic <- dat_field1 %>%

filter(Treatment == "Oxalic")

dat_field1_sub_adjuvant <- dat_field1 %>%

filter(Treatment == "Adjuvant")

\# I assume something like this was done

\# t.test(x, y, paired = TRUE, alternative = "two.sided")

t.test(dat_field1_sub_adjuvant$WashFinal, dat_field1_sub_adjuvant$WashInitial, paired = TRUE, alternative = "less")

\# This lines up with the reported values

\# Let's look at if differences between pairs are normally distributed

dat_field1 <- dat_field1 %>%

mutate(wash_delta = WashFinal - WashInitial)

hist(dat_field1$wash_delta) # The assumption of normality of differences between pairs does not hold.

\# What else can we do?

\# a) Use glmer.nb to run negative binomial mixed model, then pull out estimates and contrasts using emmeans

\# b) Or do a before-after-control-impact (BACI) analysis with negative binomial distribution (a contrast of contrasts analysis)

\# Data prep for mixed modeling

dat_field1_long <- dat_field1 %>%

rename(wash_final = WashFinal,

wash_initial = WashInitial

) %>%

pivot_longer(c(wash_final, wash_initial), names_to = c(".value", "day"), names_sep = "_") %>%

mutate(wash_r = round(wash), # Rounding this because technically negative binomial is supposed to be for count data

day = factor(day, levels = c("initial", "final"))

)

\# Mixed modeling, separately for each treatment

\# Just looking at effect of day within treatment, as with the initial paired t tests

m.2 <- glmer.nb(wash_r ~ day + (1|Colony), data = dat_field1_long, subset = (Treatment == "Adjuvant"))

summary(m.2) # almost significant reduction, P = 0.056

\# What is the estimated percent reduction?

exp(-0.3545) # Results: The final Varroa level was 0.7 times as high after treatment as before treatment. A 30% reduction.

m.3 <- glmer.nb(wash_r ~ day + (1|Colony), data = dat_field1_long, subset = (Treatment == "Control"))

summary(m.3) # Not significant

exp(0.4639) # Results: Final Varroa level was 1.59 times as high after treatment

m.4 <- glmer.nb(wash_r ~ day + (1|Colony), data = dat_field1_long, subset = (Treatment == "Oxalic"))

summary(m.4) # Not significant

exp(-0.4339) # Results: Oxalic treated colonies had 0.65 times as many Varroa after treatment as before

\# Mixed modeling, all treatments modeled together

m.5 <- glmer.nb(wash_r ~ Treatment + day + Treatment:day + (1|Colony) , data = dat_field1_long)

summary(m.5)

m.5.0 <- glmer.nb(wash_r ~ Treatment + day + (1|Colony) , data = dat_field1_long)

anova(m.5, m.5.0) # Not a significant interaction, though fairly close (P=0.15)

\# Analysis of change over time within treatment

emmeans(m.5, ~ day | Treatment, type = "response")

\# Looking at the estimates and considering the biological significance,

\# the "oxalic" group was more impressive than the "adjuvant" group, as in the previous analysis

\# BACI analysis based on mixed model passed through emmeans

emm.5 <- emmeans(m.5, ~ day * Treatment)

pairs(pairs(emm.5, simple = "day"), simple = "Treatment")

\# No significant differences in BACI analysis

\# Pre and post Varroa infestations - Year 2 ---------

dat_field2 <- read.csv("S6_Dataset.csv")

\# Look at normality

dat_field2 <- dat_field2 %>%

mutate(wash_delta = PostMitesPer100 - PreMitesPer100)

hist(dat_field2$wash_delta) # Graphically, this looks reasonably close to normally

\# distributed - perhaps because mite loads are closer to zero

shapiro.test(dat_field2$wash_delta)

\# Fails test for normality, though -

\# perhaps because of the high bump in middle

\# Mixed modeling

\# Data prep for mixed modeling

dat_field2_long <- dat_field2 %>%

rename(wash_final = PostMitesPer100,

wash_initial = PreMitesPer100

) %>%

pivot_longer(c(wash_final, wash_initial), names_to = c(".value", "day"), names_sep = "_") %>%

mutate(wash_r = round(100*wash), # Multiply by 100 and round this because technically negative binomial is supposed to be for count data

day = factor(day, levels = c("initial", "final"))

)

m.6 <- glmer.nb(wash ~ Treatment + day + Treatment:day + (1|Colony) , data = dat_field2_long)

summary(m.6)

m.6.0 <- glmer.nb(wash ~ Treatment + day + (1|Colony) , data = dat_field2_long)

anova(m.6, m.6.0) # Not a significant interaction, though fairly close (P=0.22)

\# Analysis of change over time within treatment

emmeans(m.6, ~ day | Treatment, type = "response")

\# Looking at the estimates and considering the biological significance,

\# the "oxalic" group was more impressive than the "adjuvant" group

\# BACI analysis based on mixed model passed through emmeans

emm.6 <- emmeans(m.6, ~ day * Treatment)

pairs(pairs(emm.6, simple = "day"), simple = "Treatment")

\# No significant differences in BACI analysis

\# Analysis of mite fall using repeated measures -----------------

dat_drop <- read.csv("S5_Dataset_edited_colnames.csv")

names(dat_drop)

dat_drop <- dat_drop %>%

mutate(drop_cumul = drop_0 + drop_2 + drop_4 + drop_7 + drop_14 + drop_21,

drop_0to4 = round(drop_0 + drop_2),

drop_0to6 = round(drop_0 + drop_2 + drop_4),

base = round(base),

drop_0 = round(drop_0),

drop_cumul = round(drop_cumul),

seams.x.wash = SeamsInitial*WashInitial

)

hist(dat_drop$drop_cumul, breaks = 10)

\# Negative binomial is what I would a priori go for, and it looks like a good distribution

m.7.norm <- lm(drop_cumul ~ Treatment + base, data = dat_drop)

hist(resid(m.7.norm), breaks = 10)

shapiro.test(resid(m.7.norm)) # Residuals are not statistically significantly non-normal

plot(m.7.norm) # As expected, the QQ plot is a little ugly

m.7 <- glm.nb(drop_cumul ~ Treatment + base, data = dat_drop)

summary(m.7)

m.7.0a <- glm.nb(drop_cumul ~ Treatment, data = dat_drop)

anova(m.7, m.7.0a) # Results: "baseline mite drop" was highly significant (P=0.010)

m.7.0b <- glm.nb(drop_cumul ~ base, data = dat_drop)

anova(m.7, m.7.0b) # Results: Treatment was highly significant (P<0.001)

\# Use emmeans to

\# a) make point estimates for "cumulative Varroa drop" for each treatment

emmeans(m.7, ~ Treatment, type = "response") # Confidence intervals also provided

\# These predictions are predicated on setting the baseline mite drop equal to the average baseline mite drop

\# b) Pairwise comparisons between treatments, Tukey-corrected

emmeans(m.7, pairwise ~ Treatment, type = "response")$contrasts

\# Results: The adjuvant group had 1.2 times as much cumulative mite drop,

\# though not statistically significant. This may still be worth reporting.

\# inspect model fit

plot(m.7) # Looks fine

\# Analyze just first X days

\# First 2 days

m.8 <- glm.nb(drop_0 ~ Treatment + base, data = dat_drop)

summary(m.8)

emmeans(m.8, ~ Treatment, type = "response")

emmeans(m.8, pairwise ~ Treatment, type = "response")$contrasts

6. PLOS authors have the option to publish the peer review history of their article ([what does this mean?](https://urldefense.com/v3/__https:/track.editorialmanager.com/CL0/https:*2F*2Fjournals.plos.org*2Fplosone*2Fs*2Feditorial-and-peer-review-process*23loc-peer-review-history/1/010f0195c64aba9d-fbfb0e70-433f-4c2e-89b6-67bcac0a0374-000000/Cr39LEWr2Sx0nvCFtB3i4hziwUsYYna0DRv5DmPEelI=203__;JSUlJSUl!!KGKeukY!05clY3oYy9VZII8X0ywERfkaLD4WKXhbie78VydOqFRkDFgVlMtoa-U4DEnowUh_qmOK2jNlgaxe-svQ4jlu$)). If published, this will include your full peer review and any attached files.


If you choose “no”, your identity will remain anonymous but your review may still be made public.


**Do you want your identity to be public for this peer review?** For information about this choice, including consent withdrawal, please see our [Privacy Policy](https://urldefense.com/v3/__https:/track.editorialmanager.com/CL0/https:*2F*2Fwww.plos.org*2Fprivacy-policy/1/010f0195c64aba9d-fbfb0e70-433f-4c2e-89b6-67bcac0a0374-000000/SYaMbTWioA2XNcHBbEutWN_cP9xu_0npus0149QLyWc=203__;JSUl!!KGKeukY!05clY3oYy9VZII8X0ywERfkaLD4WKXhbie78VydOqFRkDFgVlMtoa-U4DEnowUh_qmOK2jNlgaxe-rciA05Q$).

Reviewer #1: No

Reviewer #2: No

[NOTE: If reviewer comments were submitted as an attachment file, they will be attached to this email and accessible via the submission site. Please log into your account, locate the manuscript record, and check for the action link "View Attachments". If this link does not appear, there are no attachment files.]

While revising your submission, please upload your figure files to the Preflight Analysis and Conversion Engine (PACE) digital diagnostic tool, [https://pacev2.apexcovantage.com/](https://urldefense.com/v3/__https:/track.editorialmanager.com/CL0/https:*2F*2Fpacev2.apexcovantage.com*2F/1/010f0195c64aba9d-fbfb0e70-433f-4c2e-89b6-67bcac0a0374-000000/4bxOuXUs_vhDmMuWDDlNjGYIFmNt37B4g3UpXsa4WP0=203__;JSUl!!KGKeukY!05clY3oYy9VZII8X0ywERfkaLD4WKXhbie78VydOqFRkDFgVlMtoa-U4DEnowUh_qmOK2jNlgaxe-oUd2XX_$). PACE helps ensure that figures meet PLOS requirements. To use PACE, you must first register as a user. Registration is free. Then, login and navigate to the UPLOAD tab, where you will find detailed instructions on how to use the tool. If you encounter any issues or have any questions when using PACE, please email PLOS at [figures@plos.org](mailto:figures@plos.org). Please note that Supporting Information files do not need this step.
